# Modeled predictions of human-associated and fecal-indicator bacteria concentrations and loadings in the Menomonee River, Wisconsin using in-situ optical sensors

**DOI:** 10.1371/journal.pone.0286851

**Published:** 2023-06-08

**Authors:** Peter L. Lenaker, Steven R. Corsi, Laura A. De Cicco, Hayley T. Olds, Debra K. Dila, Mari E. Danz, Sandra L. McLellan, Troy D. Rutter

**Affiliations:** 1 U.S. Geological Survey, Upper Midwest Water Science Center, Madison, Wisconsin, United States of America; 2 School of Freshwater Sciences, University of Wisconsin-Milwaukee, Milwaukee, Wisconsin, United States of America; United States Environmental Protection Agency, UNITED STATES

## Abstract

Human sewage contamination of waterways is a major issue in the United States and throughout the world. Models were developed for estimation of two human-associated fecal-indicator and three general fecal-indicator bacteria (HIB and FIB) using in situ optical field-sensor data for estimating concentrations and loads of HIB and FIB and the extent of sewage contamination in the Menomonee River in Milwaukee, Wisconsin. Three commercially available optical sensor platforms were installed into an unfiltered custom-designed flow-through system along with a refrigerated automatic sampler at the Menomonee River sampling location. Ten-minute optical sensor measurements were made from November 2017 to December 2018 along with the collection of 153 flow-weighted discrete water samples (samples) for HIB, FIB, dissolved organic carbon (DOC), and optical properties of water. Of those 153 samples, 119 samples were from event-runoff periods, and 34 were collected during low-flow periods. Of the 119 event-runoff samples, 43 samples were from event-runoff combined sewer overflow (CSO) influenced periods (event-CSO periods). Models included optical sensor measurements as explanatory variables with a seasonal variable as an interaction term. In some cases, separate models for event-CSO periods and non CSO-periods generally improved model performance, as compared to using all the data combined for estimates of FIB and HIB. Therefore, the CSO and non-CSO models were used in final estimations for CSO and non-CSO time periods, respectively. Estimated continuous concentrations for all bacteria markers varied over six orders of magnitude during the study period. The greatest concentrations, loads, and proportion of sewage contamination occurred during event-runoff and event-CSO periods. Comparison to water quality standards and microbial risk assessment benchmarks indicated that estimated bacteria levels exceeded recreational water quality criteria between 34 and 96% of the entire monitoring period, highlighting the benefits of high-frequency monitoring compared to traditional grab sample collection. The application of optical sensors for estimation of HIB and FIB markers provided a thorough assessment of bacterial presence and human health risk in the Menomonee River.

## Introduction

Human sewage contamination of recreational waterways is a potential health concern, especially in urban waterways throughout the United States and world. Sources of sewage contamination include sanitary and combined sewer overflows, as well as leaking sewage conveyance systems caused by aging infrastructure and sewer misconnections, which can lead to contamination in the stormwater system and receiving waterways [1,2]. A total of 1,448 water reclamation facilities (WRFs) in the United States and Canada discharge 4.8 billion gallons (18 billion liters) of treated effluent to the Great Lakes basin each day [3]. However, a large volume of untreated sewage leaks from the conveyance system before reaching the wastewater reclamation facility. The U.S. Environmental Protection Agency (EPA) compiled exfiltration studies that included measured, computed, and estimated results from the United States, Europe, and Asia that indicated exfiltration in different systems varied from 8–56% of untreated sewage flows [4]. In addition to pathogenic bacteria and viruses [5], sewage can contain various types of contaminants, including nutrients, metals, pharmaceuticals, hormones, and toxic compounds [6–8]. The quantity, timing, and location of sewage contamination in a receiving waterway may be influenced by several factors, including the age and condition of the sanitary system, sources such as combined sewer overflows (CSOs) and sanitary sewer overflows within the drainage basin, misconnected sanitary sewers, urban hydrology dynamics, and the level of groundwater infiltration and stormwater inflow (I & I) to sanitary sewers that strain the sanitary sewer system.

Traditionally, fecal indicator bacteria (FIB) such as *E*. *coli*, enterococci, and fecal coliforms [9,10] and ammonia [11,12] have been used as indicators of fecal contamination in recreational waters; however, these indicators are not specific to human sewage. There are more specific indicators of human fecal contamination that have been used, including human-associated indicator bacteria (HIB) such as human *Bacteroides* and human *Lachnospiraceae* [13,14], pharmaceuticals and personal care products [15], and human pathogens [16,17]. Although these are effective indicators of human sewage contamination, they are also costly to measure, can take weeks to analyze, and are often not present or present at concentrations too low in sewage to confidently quantify the contamination in the environment. Thus, a human-specific, affordable, and efficient method would be valuable for detecting sewage contamination in receiving waters. Dissolved organic matter (DOM) has utility as an indicator of sewage contamination because sewage has a different DOM composition than natural waters, typically having elevated tryptophan and fulvic-like fluorescence intensities [18–20]. A rapid method for measuring DOM, highlighting the DOM changes in natural waters, could serve as a surrogate measure of sewage contamination. Fluorescence spectroscopy has been used as a method for rapid characterization of DOM from freshwater systems [21–26]. Real-time field-based sensors that target excitation/emission fluorescence wavelengths (e.g., tryptophan-like fluorescence or fluorescent dissolved organic matter (FDOM)) are commercially available, relatively inexpensive, and can be used to rapidly characterize portions of the DOM pool [27]. Municipal utilities responsible for illicit discharge detection and elimination (IDDE) programs would benefit from the further development and refined application of real-time fluorescence sensors specifically for the evaluation of human sewage contamination. Previous work has shown that there is potential for optical sensors to provide an indication of sewage presence and magnitude in natural waters [25,26,28–30]; however, there remain significant gaps in understanding the practical use in field settings such as relations of field deployable optical sensor signals to sewage presence with an unambiguous marker (such as human *Bacteroides*), uncertainties involved in these relations, reliability of sensors in long-term deployments, and transferability of optical sensor-to-sewage relationships among watersheds. Continuous fixed-point deployment of such optical fluorescent sensors, along with water collection and analysis of HIB when coupled with flow measurements will help to expand the information regarding the utility of optical sensors and fill gaps in knowledge for techniques to estimate sewage concentrations and loads to receiving water bodies.

The overall goal of this study was to use optical sensor technology to characterize the dynamics of non-effluent human sewage contamination continuously on a fine time scale in the Menomonee River. Specific objectives are as follows: 1) implement an in-situ sensor package comprised of commercially available off the shelf optical fluorometer sensors to measure specific optical properties of water, 2) collect water samples for HIB and FIB concurrently with measurements of optical properties of water during all seasons and throughout different hydrologic conditions 3) use resulting optical data to develop surrogate regressions for estimation of magnitude, and variability of HIB and FIB on a fine time scale and determine frequency of exceedance of primary-contact recreation standards, 4) estimate the proportion of sewage contamination within the stream as determined by estimation of HIB; and 5) estimate continuous loadings of sewage contamination in the stream.

## Methods

### Site selection and sample collection

**Fig 1:**
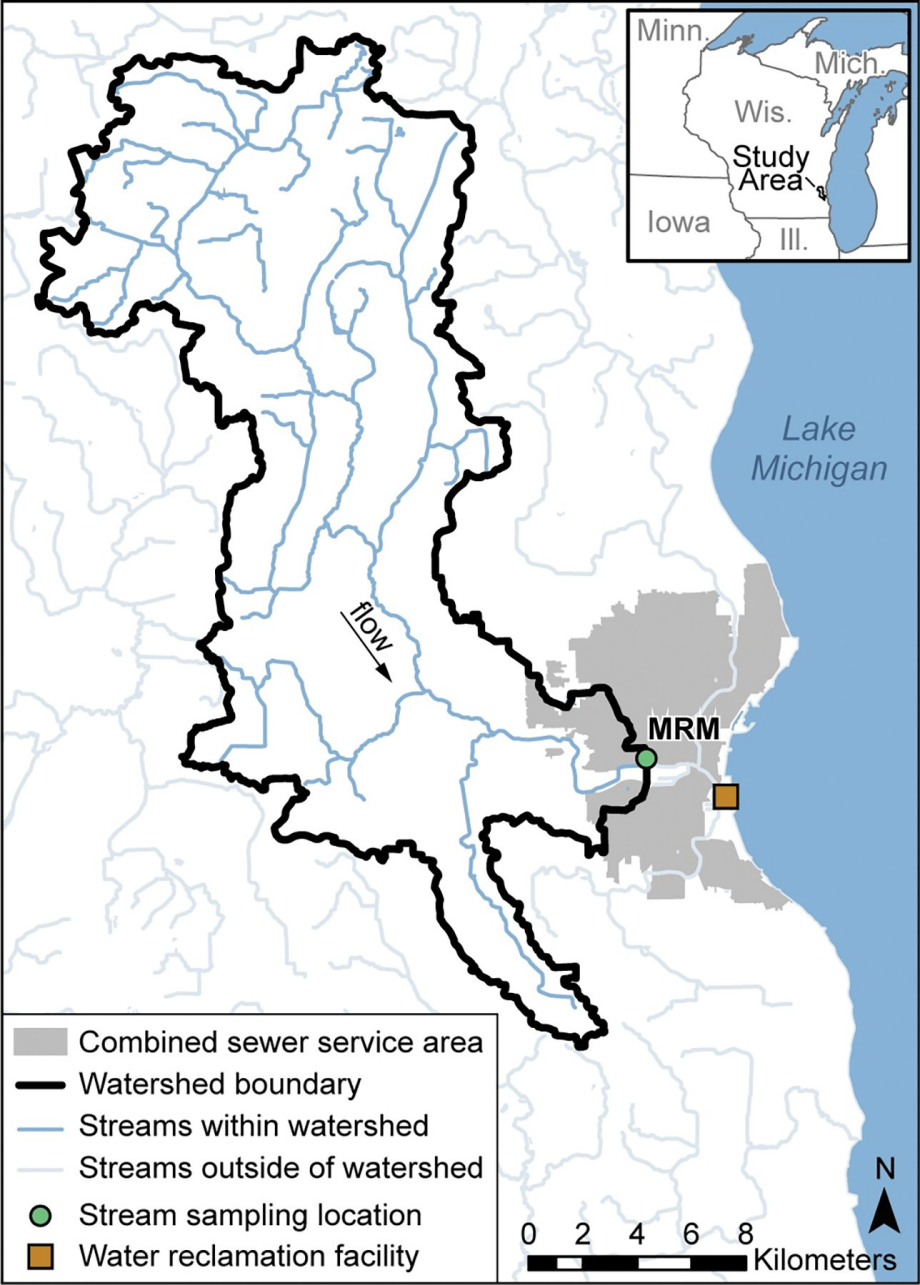
Study area map with sample locations. Stream and water reclamation facility (WRF) influent sampling locations, and the Menomonee River watershed (thick solid black line), Milwaukee, Wisconsin [31]. The shaded gray region is the Milwaukee combined sewer service area boundary where CSO discharges can occur [32], blue lines are streams [33], and gray lines in inset map are political boundaries [34]. [MRM, Menomonee River at 16th Street at Milwaukee, USGS gage 04087142].

The study was conducted from November 2017 to December 2018. Samples were collected at the Menomonee River at 16^th^ St. at Milwaukee, Wisconsin, U.S. Geological Survey (USGS) station identification number, 04087142 (hereafter referred to as MRM). The sampling location was along the south bank of the Menomonee River 1.15 miles (1.85 kilometers) upstream from the confluence with the Milwaukee River. The watershed draining to MRM was chosen because it contained no upstream water reclamation facility (WRF) effluent locations, allowing the evaluation of contamination from illicit discharges and imperfect sanitary conveyance infrastructure. Additionally, the sampling location was within the combined sewer service area and periodically received discharge from Combined Sewer Overflows (CSO) (Fig 1).

Water samples were collected monthly between December 2017 and December 2018 during periods of low flow and during periods of increased flow from rainfall and snowmelt. Samples were analyzed for three FIBs, two HIBs, dissolved organic carbon (DOC), and optical properties of water. Analysis of FIB included culture and quantitative polymerase chain reaction (qPCR) measurements of enterococci (EN) and *Escherichia coli* (*E*. *coli*, EC) and culture measurements for fecal coliforms (FC). Analysis of HIB included qPCR measurements of human *Bacteroides* (HB) and human *Lachnospiraceae* (L3). Optical properties of water included fluorescence and absorbance spectral analysis.

Multiple discrete samples were collected during each sampling event to represent variability throughout the runoff hydrograph or during a 24-h low-flow period. Discrete samples were collected using a refrigerated automatic sampler and Teflon-lined polyethylene sample tubing (model 3700R, Teledyne Isco, Inc. Lincoln, NE). An alternating 24-bottle configuration was used to allow for sequential sample collection of 300 ml samples for optical properties and DOC (round glass bottle) and bacteria (autoclaved wedge-shaped polypropylene bottle) for a potential 12 samples in each bottle type. Four to six samples were collected during individual 24-h low-flow sampling periods, and six to eleven samples were collected during individual runoff events, varying by flow response and duration of event.

Human sewage influent from one WRF was sampled monthly throughout the year to equally represent sewage influent in each season (Fig 1). A total of twelve samples were collected for FIB (qPCR only), HIB, DOC, and optical properties. One sample, August 2018, was not analyzed for FIB and HIB. WRF flow-weighted 24-hour composite influent samples were collected manually in a location prior to WRF influent mixing with recycled internal WRF streams. An aliquot from each 24-hour influent sample was composited over a five-day period. WRF influent samples were filtered within 24–36 h after the last daily sample was added to the composite. Additional information on WRF influent sample collection can be found in S1 Appendix.

### Laboratory methods

#### Fecal- and human-associated indicator bacteria

Immediately upon arrival to the laboratory, a volume of 200 mL from each surface water sample was filtered through a 0.22 μm pore mixed cellulose ester filter (47 mm diameter; Millipore, Billerica, Massachusetts) with the exception of three samples that had a sample volume of 100 mL filtered. Filters were then folded and placed in 2 mL screw-cap vials and immediately stored at -80°C until DNA extraction. Additional details for computing the limit of quantification based on the volume of water filtered are presented in S1 Appendix.

For qPCR analyses, DNA was extracted from frozen filters using MPBIO FastDNA® SPIN Kit for Soil (MP Biomedicals, Santa Anna, California) within six months of filtering. Prior to extraction, salmon testes DNA was added to extraction buffer according to USEPA Method 1611 [35]. In previous studies using this method, extraction efficiencies were determined to be 46.5% ±3% [36] and inhibition studies demonstrated no inhibition of qPCR in river water [36] or stormwater [2] samples. A subset of study samples (n = 14, 9% of samples) were tested for inhibition by these methods, and no inhibition was noted. Samples were analyzed by qPCR for human *Bacteroides* (HB, HF183) [37], human *Lachnospiraceae* (L3, Lachno3) [38], enterococci (EN) [35], and *Escherichia coli* (EC) [2] assays as described previously. Field blanks (n = 2) were extracted with study samples and served as controls for field apparatus, filtering apparatus, and extraction controls. All field and qPCR blanks (reactions without DNA template added) (included on every analytical batch) were negative; however, one field blank sample had detections of cultured enterococci (1.0 CFU/100 mL) and fecal coliform (5.0 CFU/100 mL). Both of these values are substantially below all environmental results. Additional information on quality assurance sample collection can be found in S1 Appendix.

Assays for HB, L3, EN, and EC were carried out by Applied Biosystems StepOne Plus Real-Time PCR System Thermal Cycling Block (Applied Biosystems; Foster City, California) with Taqman hydrolysis probe chemistry, and assay conditions followed manufacturer’s instructions. Inter-run controls consisted of environmental samples that were previously run on a minimum of three separate occasions. Concentrations were determined from standard curves. The HB assay standard curve slope = -3.36, y = 37.11, efficiency 98.62%, r^2^ = 0.998; the L3 assay standard curve slope = -3.45, y = 38.38, efficiency 94.99%, and r^2^ = 0.996; the EN assay standard curve slope = -3.43, y = 39.77, efficiency 95.82%, and r^2^ = 1.00; the EC assay standard curve slope = -3.48, y = 39.27, efficiency 93.76%, and r^2^ = 0.999. Additional details for DNA extraction, qPCR assays, and culture-based analysis are provided in S1 Appendix. Culture-based results are reported in colony-forming units per 100 ml (CFU/100 mL) and qPCR results are reported in units of copy number per 100 ml (CN/100 mL).

#### Dissolved Organic Carbon (DOC) and Fluorescence and Absorbance measurement

Stream water from the 300 mL glass bottle was used for dissolved organic carbon (DOC) analysis and simultaneous analysis of fluorescence and absorbance (optical analysis). DOC and optical analysis were completed at the U.S. Geological Survey Upper Midwest Water Science Center in Madison, Wisconsin. Environmental samples for DOC and optical analyses were filtered (Whatman glass microfiber syringe filters, pore size 0.45 μM) into two pre-combusted 40 ml amber glass vials. Samples were stored in the dark at 4°C until sample analysis. All samples for optical analysis were analyzed within 5 days of collection, and DOC samples were analyzed within 14 days of collection [39].

DOC samples were analyzed using a Shimadzu TOC-V_CSH_ analyzer, coupled with the Shimadzu ASI-V auto sampler. The non-purgeable organic carbon (NPOC) analysis method was employed, in which samples were sparged with 2 M HCl to remove all inorganic carbon prior to combustion. Based on a 1 mg C L^-1^ DOC standard (Organic Carbon standard, RICCA Chemical Company, Arlington, Texas) measured from 2017 to 2018, a method detection limit (MDL) of 0.108 mg C L^-1^ was computed.

Fluorescence excitation-emission matrix (EEM) and absorbance scan measurements were performed using a Jobin Yvon Aqualog benchtop Spectrofluorometer (HORIBA Scientific, Piscataway, New Jersey). EEMs were generated using excitation (ex) wavelengths of 240–800 nm at intervals of 3 nm and emission (em) wavelengths of 247–827 nm at intervals of 2.33 nm with an integration time of 1 s and a CCD gain set at medium. For absorbance at each wavelength and select fluorescence signals, the minimum reporting limit (MRL) was calculated as the mean absorbance, or fluorescence signal, for the field blank samples (ultra-pure 18.2 megohm water) plus three standard deviations. The MRLs for the select absorbance and fluorescence signals for the present study are available online [40]. Additional details related to DOC and fluorescence and absorbance analysis can be found in S1 Appendix.

Two quality assurance field blank samples were collected; DOC and optical property results were below the method detection levels. Additional information on quality assurance sample collection can be found in S1 Appendix.

### Deployed sensors

**Fig 2:**
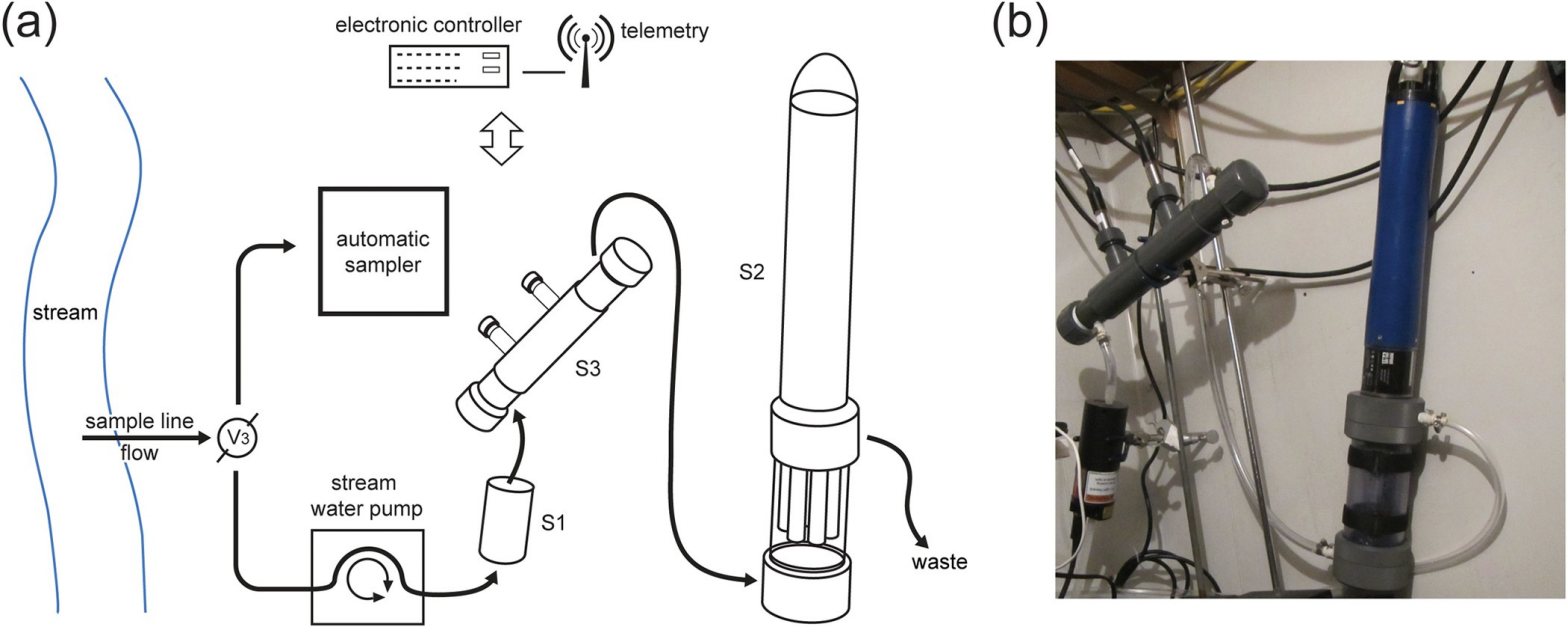
Schematic and photo of flow-through system. (A) Diagram of the unfiltered custom-designed flow-through sensor and automatic sampler system and (B) photo of the three different optical sensor platforms deployed for the duration of the study. The S1 meter included three sensors selected to characterize different unique areas of the excitation-emission spectra. The S2 meter included water temperature, turbidity, and fluorescent dissolved organic matter (fDOM) sensors. The S3 meters included individual sensors for CDOM and tryptophan-like fluorescence. Photograph by Peter L. Lenaker, USGS. [V3, valve for directing flow to the sensor manifold and automatic sampler].

Three commercially available optical sensor platforms were included in a custom flow-through system at MRM that was designed to deliver unfiltered water for real-time measurement of turbidity, water temperature, and six unique measurements of optical properties (Fig 2). A WET Labs WETStar FDOM flow-through 3-channel fluorometer (WET Labs, Inc, Sea Bird Scientific, Philomath, Oregon) included three measurement channels to characterize different unique areas of the excitation-emission spectra (referred to as S1-CF, S1-A, and S1-T) (Table 1). A YSI EXO2 with an EXO2 flow cell and anti-fouling central wiper was deployed with temperature, turbidity, and fluorescent dissolved organic matter (FDOM, referred to as S2-F) sensors (YSI Incorporated, Yellow Springs, Ohio). Lastly, two Turner Designs Cyclops-7 chromophoric dissolved organic matter (CDOM)/FDOM (abbreviated S3-CF) and Tryptophan-like fluorescence (abbreviated S3-T) open-faced sensors were installed into a custom-designed manifold (Turner Designs, San Jose, California; Table 1). Sensors from the three manufacturers (S1, S2, and S3) represented a range of excitation and emission wavelengths (A, T, CF, and F, Table 1, S1 Fig in S1 Appendix). Additional details related to deployment, maintenance, and operation of the optical sensors and the automated sampler water sample collection are presented in S1 Appendix.

**Table 1:** Description of in-situ deployed fluorescing dissolved organic matter (FDOM) and tryptophan-like fluorometers, and explanatory variable abbreviation.

Sensor	Excitation (nm)^a^	Emission (nm)^a^	Explanatory Variable Abbreviation
WETLabs WETStar Channel 1	310 (±10)	452 (±22)	S1-CF
WETLabs WETStar Channel 2	280 (±7)	452 (±22)	S1-A
WETLabs WETStar Channel 3	280 (±7)	350 (±20)	S1-T
YSI FDOM	365 (±5)	480 (±40)	S2-F
Turner Cyclops-7 CDOM	325 (±60)	470 (±30)	S3-CF
Turner Cyclops-7 Tryptophan	280 (±5)	350 (±28)	S3-T

^a^Excitation-emission wavelengths (± bandpass value) given in nanometers (nm).

### Data analysis and processing

The potential for L3 and HB assays to cross-react with samples from other animal species (e.g. dog, cat, and raccoon) does exist [38]. Therefore, concentrations from individual human-associated indicator bacteria (L3 and HB) were summed to compute a “sum of human-associated indicator bacteria marker” (sHM) for each sample to provide higher resolution and specificity for assessing human fecal contamination. Streamflow (MRM, 04087142), human-associated indicator bacteria (HIB), fecal indicator bacteria (FIB), dissolved organic carbon (DOC), and uncorrected continuous time-series sensor data from the present study are archived in the U.S. Geological Survey National Water Information System (NWIS) [41,42]. Additional details for accessing streamflow, HIB, FIB, and uncorrected continuous time-series data are provided in S1 Appendix. Select parameters from laboratory Aqualog absorbance and fluorescence measurements, along with computed one-hour mean streamflow and the corrected continuous time-series data for the optical sensors for the present study are available online [40].

Raw in-situ field sensor time-series data were foul- and drift-corrected following approved U.S. Geological Survey techniques and methods [43,44]. Briefly, foul corrections were applied based on the difference between the before cleaning and after cleaning sensor readings while in organic free reagent water (ultra-pure 18.2 megohm water) (S2 Table in S1 Appendix). Drift corrections were determined through measurements across a range of quinine sulfate concentrations for S1-CF, S1-A, S2-F, and S3-CF FDOM sensors and across a range of tryptophan concentrations for S1-T and S3-T tryptophan-like sensors prior to field deployment (beginning of study) and after field deployment (end of study). Drift corrections by sensor were as follows, S1-CF, 260%; S1-A, 86.5%; S2-F, -6.6%; S3-CF none; S1-T, 106%; and S3-T none.

Examination of the relation between sensor-measured values and laboratory-measured values indicated that temperature and turbidity effects on FDOM results likely influenced final values. Data were therefore adjusted using temperature and turbidity based on established methods: This included a linear relationship with the difference in field-measured temperature and the temperature at which samples were analyzed in the laboratory [45,46] and a non-linear exponential regression relationship with turbidity [47,48]. The relationship of measurements from sensor S3-CF with temperature changed on March 1, 2018 after this sensor malfunctioned and a new sensor was installed: before this time, a relationship with temperature was not evident, but after this time, the relationship with temperature was consistent with Watras et al., 2011 [45]. Examination of the ratio of the sensor-measured values to the laboratory-measured values for S1-CF and S1-A did not uncover temperature and turbidity effects. For these two sensors, data were only converted from original field-measured units (millivolts) to laboratory-measured units (Raman Units) by applying an offset and slope estimated using least squares regression. Sensor values measured for S1-T and S3-T had an erratic relationship with laboratory-measured values that changed unpredictably over time, were considered to be unreliable, and were not used further in this study.

Statistical significance was determined with a pair-wise Wilcoxon rank sum test with corrections for multiple comparisons for FIB and HIB concentrations [49]. Statistical significance for pairwise comparisons of individual human markers and fecal bacteria and the three different flow conditions was determined using generalized linear (binomial) models, in addition to evaluating if significant relations between like field sensor variables exist using Kendall’s *tau* statistic [50–52]. In all cases, significance was evaluated at the 5% two-sided risk level.

### Regression modeling

Regression models were developed to explore the relationship between the optical signals (S1-CF, S1-A, and S2-F), turbidity, mean one-hour flow from the MRM sample location, seasonal interaction variables (explanatory variables), and three FIB, two HIB, and one sum of HIBs (sHM) bacteria marker measurements (response variables). Sensors S2-F and S3-CF were correlated (r = 0.896). To avoid overfitting the model, only one of these signals was used in model development: S2-F was chosen over S3-CF because S2-F had a more complete data record than S3-CF, and the data record for S3-CF required more extensive post-monitoring adjustments to account for biofouling that occurred in the absence of a wiper system. Sensor S2-F had a wiper system that minimized biofouling.

The model selection process included several steps to minimize prediction error. Given the prevalence of censored data in the response variable results (bacteria), survival regression with an assumed Gaussian distribution for the response variables was used for development of models. The response data were log-base 10 transformed. Explanatory variables for the optical sensors, S1-CF, S1-A, and S2-F, also included seasonal variables (sine and cosine of (Julian day/365.25)2π) as interaction terms to account for the potential of background dissolved organic matter changes through the year, consistent with a previous effort that has demonstrated this approach to be effective [53]. Results of initial regressions with the full data set suggested that the relation between optical properties and bacteria changed during periods of CSO discharge to the stream. To account for this, regressions were developed three ways: with only CSO-influenced data, with data that did not include CSO influence (low-flow and event-runoff), and with all data combined. Five-fold cross validation repeated 50 times was conducted to estimate predictive accuracy. The median normalized root mean squared error in prediction (NRMSEP) for the 50 model fits was used to compare among models with different explanatory variable combinations. A two-step selection process was used to select the combination of explanatory variables in the final model for each response variable: 1) lowest median NRMSEP; 2) if various models had a median NRMSEP within three percent of each other, the model with the minimum number of explanatory variable terms was chosen [53]. A model was developed for each response variable (eight response variables) for the three different CSO influence conditions for a total of 24 different model scenarios. Bacteria estimation from models included back-transformation from log-space and correction using the smearing estimator [54]. Statistical modeling was conducted in the R statistical software [52] using the survival package to conduct linear modeling and the cvTools package for facilitating cross validation routines [55,56]. A model archive which included input data used to develop ordinary least squares regression and linear mixed effect models is publicly available [40].

### Model estimations: Concentration, loads, and proportion of sewage

The non-CSO model was used for estimating concentrations except during six CSO discharge periods (S6 Table in S1 Appendix) where the CSO model was applied. Bacteria loads, expressed as counts of bacteria, were computed by multiplying the estimated concentrations by stream-water volume. Stream-water volume was computed by integrating 10-minute discharge measurements from the USGS MRM location. Daily loads of bacteria were computed by multiplying estimated 10-minute bacteria concentrations by MRM streamflow and integrating these values for a total load each day.

To estimate the quantity of raw human sewage contamination in the stream, an estimate of the proportion of sewage was calculated for the human-associated bacteria markers (HB, L3, sHM). The proportion of sewage estimates are unit-less and represent an estimate of sewage within the stream. The proportion of sewage for each bacteria marker was computed by dividing the estimated concentration for each bacteria marker by the mean concentration of the bacteria marker in WRF sewage influent. A mean annual proportion of sewage was computed for sHM by taking the sum of daily sHM loads and dividing by the sum of daily MRM streamflow to get the average annual sHM estimated stream concentration, which was then divided by the average WRF influent concentration. Using the proportion of sewage and MRM streamflow, an estimate of the annual volume of sewage delivered to the MRM sampling location was computed.

### Comparison to water quality benchmarks

Bacteria concentration estimates resulting from this study can be used to evaluate the potential for human health risk. The Menomonee River in Milwaukee, Wisconsin flows into the Milwaukee River estuary and can have a negative impact on these downstream recreational waters. First, recreational water quality criteria have been developed by the U.S. Environmental Protection Agency (U.S. EPA) for fecal indicator bacteria [10]. Second, researchers have used HB in quantitative microbial risk assessment (QMRA) to estimate risk to swimmers based on HB concentrations [36,57]. These methods are used to evaluate data from the current study.

Recreational water quality criteria for the FIB, *E*. *coli* and enterococci, have been established by the U.S. EPA for primary contact designated uses [10]. For the bacteria and markers included in the current study, there are two potential ways to apply recreational water quality criteria published by the U.S. EPA: by single sample or by geometric mean (GM) and statistical threshold value (STV) (“The waterbody GM should not be greater than the selected GM magnitude in any 30-day interval. There should not be greater than a ten percent excursion frequency of the selected STV magnitude in the same 30-day interval”) [10]. Data from the current study were evaluated with these two methods using the U.S. EPA recreational water quality criteria for a gastrointestinal (GI) illness rate of 36 individuals per 1,000 for primary contact recreators for enterococci and *E*. *coli* in fresh waters.

In addition, human-associated indicators of fecal contamination (HB) have been used to monitor human sewage impacted waters [2,38,58,59], and quantitative microbial risk assessments have been developed with these data to simulate the risk of GI illness associated with swimming using HB concentrations in two different studies that resulted in benchmark thresholds of 4,200 CN/100 mL and 7,800 CN/100 mL [34,51, respectively]. The HB benchmark value of 4,200 CN/100 mL data were derived based on the relation between six reference pathogens and HB in sewage and the associated risk of infection by the reference pathogens [57]. The HB benchmark value of 7,800 CN/100 mL was based on the relation between norovirus and HB concentrations in sewage and the associated risk of norovirus infection [36]. These benchmark differences are likely driven by the human virus concentrations in the sewage samples used in those studies, which can be highly variable across seasons [5]. Data from the current study were also evaluated by summarizing exceedances of these two benchmarks (Table 2).

**Table 2:** Recreational water quality criteria for enterococci and *E*. *coli* for an estimated illness rate of 36 per 1,000 primary contact recreators [10], and quantitative microbial risk assessment estimation of risk for an illness rate of 30 per 1,000 primary contact recreators for human *Bacteroides* [36,57].

Indicator bacteria	Analytical method	Concentration (units per 100 mL)	Application method	Model-estimated percent exceedance during monitoring period*	Reference
Enterococci	Culturable	35 cfu with 130 cfu STV	30-day GM with 10% or less STV	61%	[10]
*E*. *coli*	Culturable	126 cfu with 410 cfu STV		47%	[10]
Enterococci	Culturable	70 cfu	Single sample Beach action values	48%	[10]
*E*. *coli*	Culturable	235 cfu		34%	[10]
Enterococcus spp.	qPCR	1000 cce		96%	[10]
Human *Bacteroides*	qPCR	4200 CN	Single sample	66%	[57]
Human *Bacteroides*	qPCR	7800 CN	Single sample	58%	[36]

[* for estimating percent exceedance during the monitoring period, the CSO model was used during known CSO times and the non-CSO model during non-CSO times. GM, geometric mean; STV, statistical threshold value; cfu, colony forming units; CN, copy number; cce, calibrator cell equivalent; mL, milliliter].

## Results

### Water monitoring

**Fig 3:**
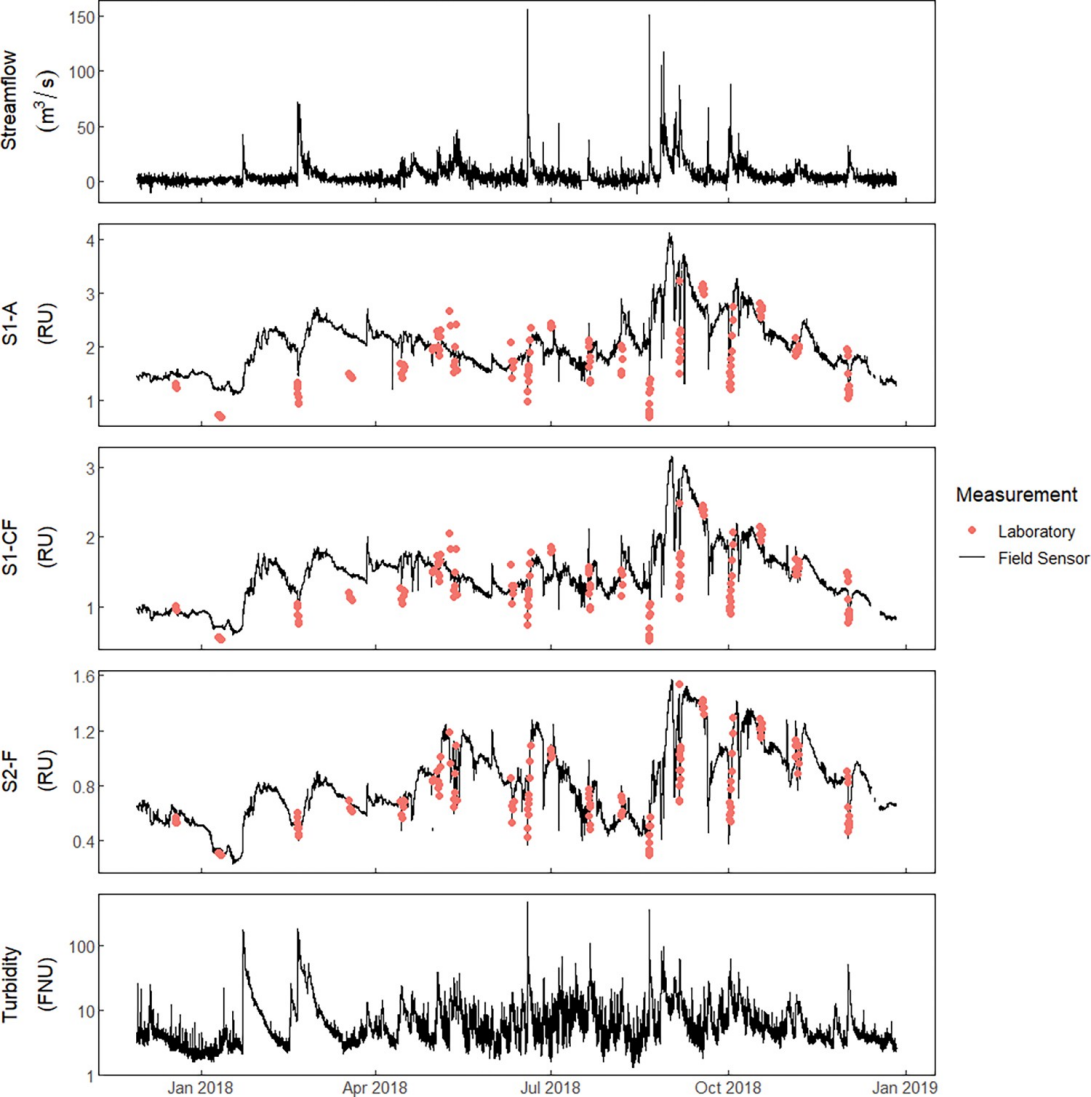
Time series for streamflow, optical sensor values, and turbidity with laboratory-measured optical properties of water. Streamflow, optical field sensor values, and turbidity are represented by black lines. Laboratory-measured optical properties of water from grab samples are represented by filled circles. [m^3^/s, cubic meters per second; RU, raman units; S1-A, excitation 280 (±7), emission 452 (±22) nanometers (nm); S1-CF, excitation 310 (±10), emission 452 (±22) nm; S2-F, excitation 365 (±5), emission 480 (±40) nm; FNU, formazin nephelometric unit].

Turbidity, water temperature, and fluorescence sensors were deployed from November 2017 to December 2018, and 153 discrete water samples were collected from December 2017 to December 2018, to evaluate real-time sensor measurements and to quantify FIB, HIB, and laboratory-measured optical properties of water. Of those 153 water samples, 119 samples were collected during event-runoff periods and 34 were collected during low-flow periods. Further, of the 119 event-runoff samples, 43 samples were collected during CSO-influenced event-runoff periods (referred to as event-CSO periods) (Fig 3, S7 Table in S1 Appendix). In addition, from December 2017 to December 2018, 12 5-day composite WRF influent samples were collected (S8 Table in S1 Appendix, S2 Fig in S1 Appendix).

Corrected time-series for field sensors S1-A, S1-CF, and S2-F, along with mean one-hour streamflow and turbidity were used as explanatory variables in the regression modeling to estimate FIB and HIB (Fig 3). Final corrected optical sensor values were positively correlated with laboratory-measured values for field sensors S1-A (r = 0.66), S1-CF (r = 0.78), and S2-F (r = 0.99) (Fig 3).

**Fig 4:**
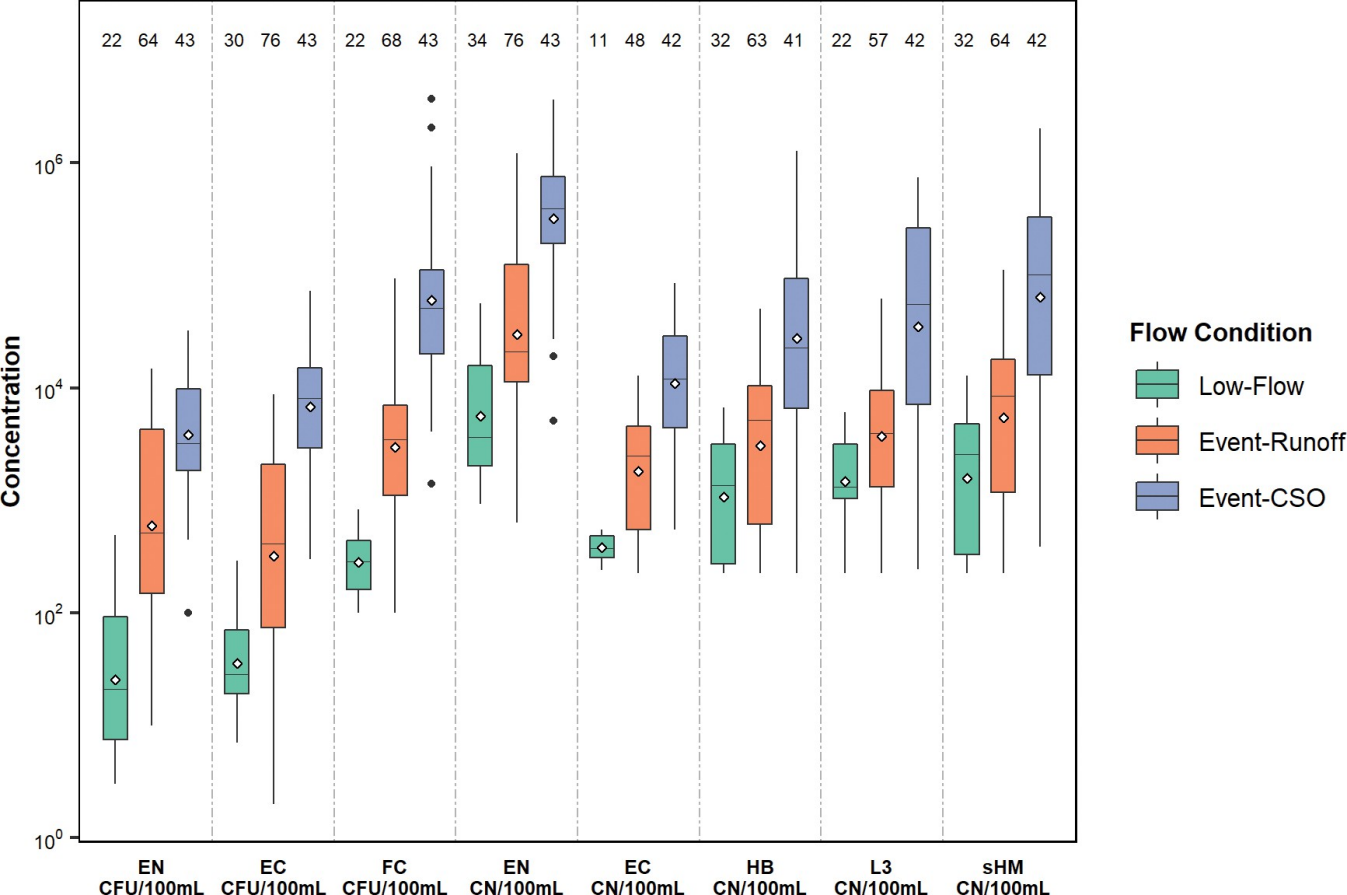
Boxplots of sample distribution for human-associated and fecal indicator bacteria during low-flow, event-runoff, and event-CSO periods for the Menomonee River at 16^th^ Street at Milwaukee, Wisconsin, December 2017-December 2018. Numbers along top of plot indicate the number of samples collected per bacteria and flow condition. The gray dashed line separates the three box plots by bacteria. Cultured bacteria markers have units of CFU/100 mL and qPCR bacteria markers have units of CN/100 mL. [Boxes, 25th to 75th percentiles; horizontal line, median; diamond, mean; whiskers, data within 1.5× the interquartile range (IQR); and circles, values outside 1.5× the IQR. [EN, enterococci; EC, *Escherichia coli*; FC, fecal coliform; HB, human *Bacteroides*; L3, human *Lachnospiraceae*; sHM, sum of human markers (HB + L3); CFU, colony forming units; CN, copy numbers].

Median concentrations of L3 and HB in stream samples were 1,100 and 1,800 CN/100 mL with detection rates of 79% and 89%, respectively (Fig 4, S7 Table in S1 Appendix). Median concentrations of qPCR EN and EC in stream samples were 31,000 and 570 CN/100 mL with detection rates of 100% and 66%, respectively (Fig 4, S7 Table in S1 Appendix). Previous reports evaluating the enterococci qPCR method suggests this assay overestimates the 23S rRNA CN [60,61]. Cultured bacteria EN, EC, and FC in stream samples had overall median concentrations of 700, 570, and 4,700 CFU/100 mL with detection rates of 93%, 97%, and 99%, respectively (Fig 4, S7 Table in S1 Appendix). The HIB (L3 and HB) and FIB (EC, EN, and FC) results indicated that median concentrations were more than an order of magnitude greater during event-CSO periods for each of the bacteria measured in this study (Fig 4, p < 0.05).

Concentrations of EN (CN/100 mL) in WRF influent samples were on average 1.5 orders of magnitude greater than the other HIB and FIB markers with all bacteria markers occurring in 100% of WRF influent samples (S8 Table in S1 Appendix). WRF influent concentrations varied less than an order of magnitude through the year except for lower observed HB and L3 concentrations for samples collected in December 2017 and March 2018 (S2 Fig in S1 Appendix).

### Optical sensor signals and bacteria–regression relationships

**Fig 5:**
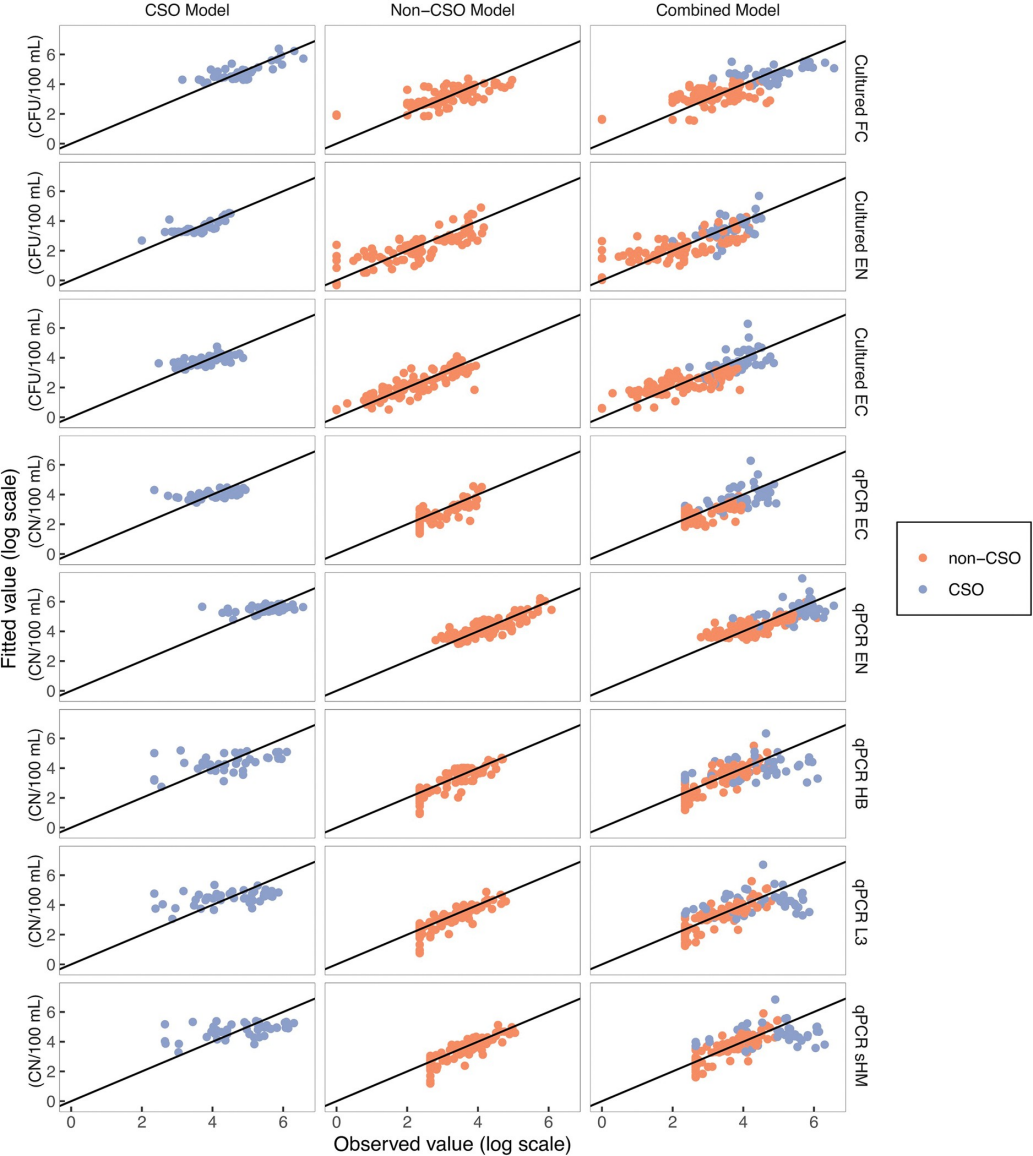
Observed fecal indicator and human indicator bacteria concentrations compared to fitted values from selected combined sewer overflow (CSO), non-CSO, and the combined data set regression models, Menomonee River, Milwaukee, Wisconsin, December 2017 to December 2018. [FC, fecal coliform; EN, enterococci; EC, *Escherichia coli*; HB, human *Bacteroides*; L3, human *Lachnospiraceae*; sHM, sum of human markers (HB + L3); qPCR, quantitative polymerase chain reaction; CFU/100 mL, colony forming units per 100 milliliters; CN/100 mL, copy number per 100 milliliter].

Initial regression modeling included all samples collected regardless of hydrologic condition and CSO influence. This resulted in poor cross-validated predictive capability for these models during CSO periods, with the exception of cultured FC and EN, compared to models developed with data that were separated by CSO and non-CSO periods (Fig 5). The average median NRMSEP (across all bacteria groups) were 10% lower for non-CSO periods and 53% higher for CSO periods than for the combined models. For this reason, the CSO and non-CSO models were used in final estimations for CSO and non-CSO time periods, respectively (Table 3, S9 Table in S1 Appendix, S3 Fig in S1 Appendix). It is anticipated that real-time predictions would use the models developed with all data because it is likely that the sensor system will not have sufficient accurate information on CSO discharge occurrence in real time to change from non-CSO to CSO models during the course of a CSO event. Estimations would need to be adjusted to use the separated CSO and non-CSO models when the CSO extent and timing is defined after the event. For this reason, all models regardless of CSO condition are presented here (Table 3). Model exploration with and without the seasonal interaction term confirmed that inclusion of the seasonal interaction term resulted in improved model fit, so this approach was used in all models.

**Table 3:** Explanatory variables and median normalized root mean squared error in prediction (NRMSEP) for regression models to estimate bacteria marker concentrations during (A) combined sewer overflow (CSO) periods, (B) non-CSO periods, and (C) all data combined periods. An “x” indicates a model variable was selected and included in the regression model.

**(A) CSO periods**	Model variables					
	Interaction with season					
Response Variable–Bacteria Marker	S1-CF	S1-A	S2-F	Turbidity	MRM Mean one-hour flow	NRMSEP median
Fecal coliform (culture)		x	x			0.88
Enterococci (culture)	x		x			0.76
*E*. *coli* (culture)		x	x		x	1.07
*E*. *coli* (qPCR)		x	x			1.17
Enterococci (qPCR)		x	x			1.18
Human *Bacteroides* (qPCR)		x	x			1.12
Human *Lachnospiraceae* (qPCR)		x	x			1.16
Sum of Human Marker (sHM)		x	x			1.16
**(B) non-CSO periods**	Model variables					
	Interaction with season					
Response Variable–Bacteria Marker	S1-CF	S1-A	S2-F	Turbidity	MRM Mean one-hour flow	NRMSEP median
Fecal coliform (culture)		x	x		x	0.80
Enterococci (culture)	x	x	x	x	x	0.70
*E*. *coli* (culture)	x	x	x	x	x	0.58
*E*. *coli* (qPCR)		x	x	x	x	0.71
Enterococci (qPCR)	x		x	x	x	0.60
Human *Bacteroides* (qPCR)		x	x	x		0.56
Human *Lachnospiraceae* (qPCR)		x	x		x	0.56
Sum of Human Marker (sHM)		x	x		x	0.53
**(C) Combined**	Model variables					
	Interaction with season					
Response Variable–Bacteria Marker	S1-CF	S1-A	S2-F	Turbidity	MRM Mean one-hour flow	NRMSEP median
Fecal coliform (culture)	x	x	x		x	0.73
Enterococci (culture)	x	x	x		x	0.67
*E*. *coli* (culture)	x	x	x		x	0.61
*E*. *coli* (qPCR)	x	x	x		x	0.67
Enterococci (qPCR)	x	x	x		x	0.66
Human *Bacteroides* (qPCR)	x	x	x		x	0.77
Human *Lachnospiraceae* (qPCR)	x	x	x		x	0.74
Sum of Human Marker (sHM)	x	x	x		x	0.73

Median NRMSEP is reported in log base 10 of the response variable. One-hour flow is a one-hour rolling mean of instantaneous five-minute flow from the USGS station, Menomonee River at 16th St. at Milwaukee, Wisconsin (MRM), 04087142. [culture, CFU/100 mL, colony forming units per 100 milliliters; qPCR, CN/100 mL, copy number per 100 milliliters; Explanatory variable abbreviations are defined in Table 1].

Models included different combinations of fluorescence sensor signals S1-CF, S1-A, S2-F with a seasonal interaction term, and turbidity (Table 3). Sensor signal S2-F was selected in all models, while S1-A was selected in all models except one for cultured enterococci (CFU/100 mL) during CSO periods, S1-CF was included in selected models for all versions of the data set, and turbidity was included in selected models for non-CSO periods (Table 3). A flow variable was included in only one of the selected models for CSO periods, all models except one for the non-CSO periods, and all models for the combined data set (Table 3).

### Estimates of bacteria concentration, sewage content, and loading

**Fig 6:**
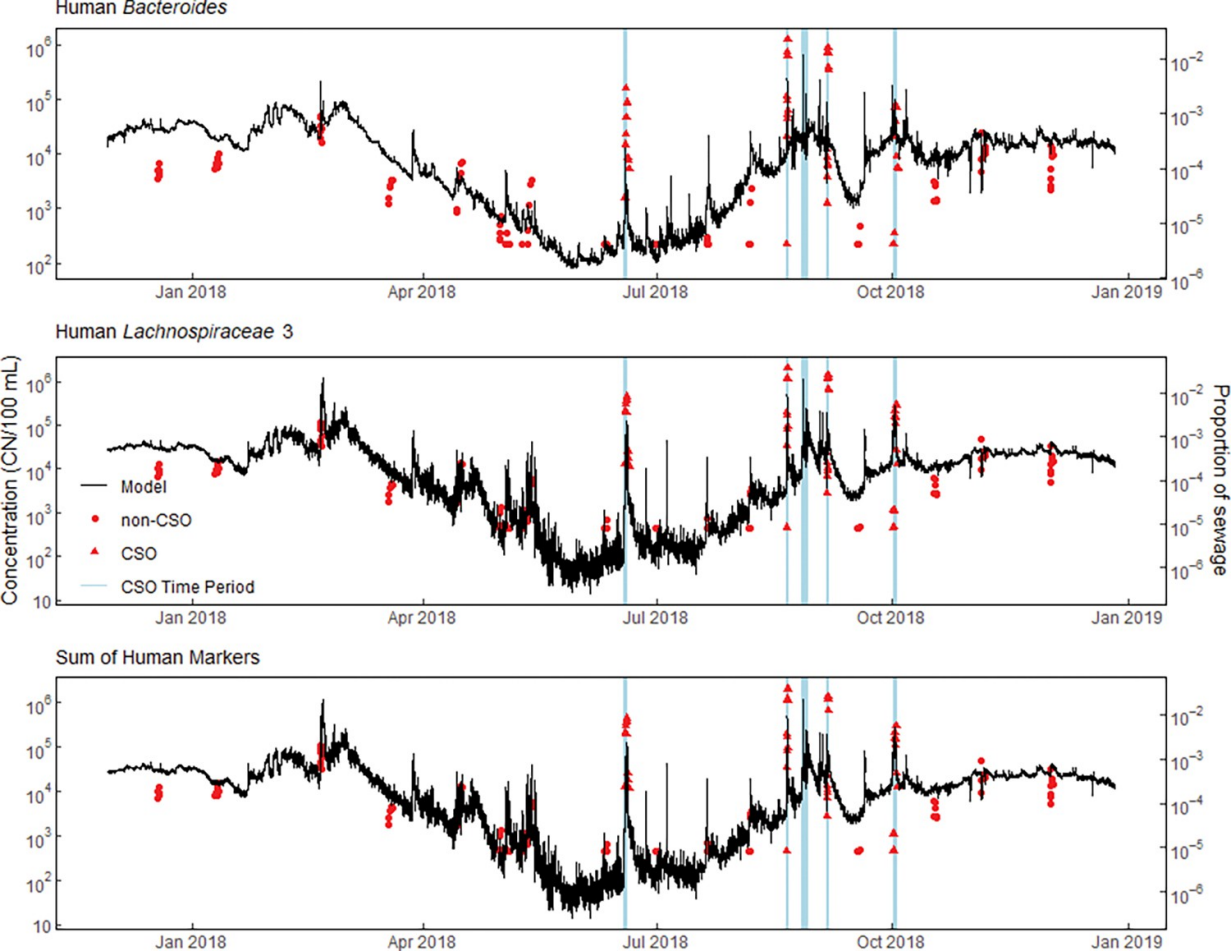
Stream sample results and model estimations of continuous human-associated indicator bacteria concentrations and proportions of sewage at 10-minute intervals for the Menomonee River in Milwaukee, Wisconsin, December 2017 to December 2018. Vertical, blue-shaded regions, represent event-CSO time periods. Predictions for all time periods were derived using the “CSO model” for CSO periods and the “non-CSO model” for non-CSO time periods. When estimated values exceed the stream sample concentration data used for model calibration, the estimations are designated as > 1.3 x 10^6^ CN/100 mL for HB, > 7.4 x 10^5^ CN/100 mL for L3, and > 2.0 x 10^6^ CN/100 mL for sHM. [CN/100 mL, copy number per 100 milliliters].

Like measured concentrations, estimated continuous concentrations for HIB and FIB markers varied over six orders of magnitude during the monitoring period, and the greatest estimated concentrations occurred during event-runoff and event-CSO periods (Fig 6, left Y-axis and S4 Fig in S1 Appendix).

The proportion of sewage in the Menomonee River was estimated using the mean WRF influent concentration for the study period and the continuous estimated concentrations for HIB markers and sHM (Fig 6, right Y-axis). The proportion of sewage present in the stream at any given 10-minute interval varied by nearly five orders of magnitude from 2.6 x 10^−7^ (0.000026%) to 0.028 (2.8%) sewage content in the stream depending on the stream conditions. The flow-weighted annual mean proportion of sewage for CSO periods were an order of magnitude greater, 0.0011 (0.11%), then during non-CSO periods 0.00034 (0.034%) for the sHM.

**Fig 7:**
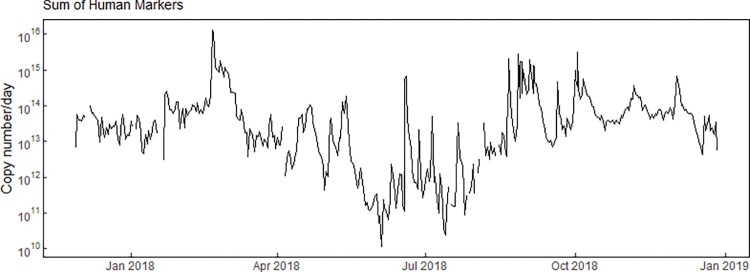
Estimates of daily sum of human markers (sHM) load (black line) for the Menomonee River in Milwaukee, Wisconsin, December 2017- December 2018. Vertical, blue-shaded regions, represent event-CSO time periods.

Estimated continuous loads for HB, L3, and sHM ranged from 10^10^ to 10^16^ copy numbers/day with substantial increases in the load resulting from the combination of increased concentrations and increased streamflow during event-runoff and event-CSO periods (Fig 7 and S5 Fig in S1 Appendix). The flow-weighted annual mean proportion of sewage was estimated at 0.00068 (0.068%) sewage content in the stream, computed as the total annual loading for sHM divided by the total annual streamflow (Fig 7). Estimated loads for the three culture-based FIB and the two qPCR FIB were similar in magnitude, ranging from approximately 10^9^ to 10^17^ copy numbers/day, also with substantial increases in the load as a result of increased streamflow and concentrations during runoff events (S5 Fig in S1 Appendix).

## Discussion

### Real-time bacteria concentration predictions

Concentrations of bacteria in streams can be highly variable on a short time scale during variable hydrologic conditions [37,58]. It is not typically practical to collect enough discrete samples to adequately represent these dynamic water quality changes given the complexity of high intensity sampling and the expense and time needed for laboratory analyses. Surrogate models developed in the current study provided a means to estimate this concentration variability over short temporal scales, incorporating variability driven by seasonal, diurnal, hydrologic, and other environmental changes.

Comparison of the continuous modeled concentrations for the study period to FIB and HB benchmarks indicated that recreational water quality criteria for the FIB, *E*. *coli*, and enterococci were exceeded 34% to 96% of the time depending on the organism and method used, and HB benchmark concentrations were exceeded 66% and 58% of the time (Table 3). This similar rate of exceedances for 4,200 and 7,800 CN/100 mL benchmark levels highlights the dynamic range of HB during contamination events where levels can increase over orders of magnitude (Fig 6). Overall, concentration exceedances were more prevalent during colder-weather months as opposed to warmer months when contact-recreation is most likely to occur. The primary periods of exceedance during the contact recreational season were during runoff events, active CSO periods, and later in the summer (Fig 6). Further, while CSO periods may be short in duration, they can contribute to periods of exceedance during non-CSO runoff events because FIB and HIB can become buried in sediments, extending survival by months due to protection from ultraviolet light and viral and bacterial predation [62].

Comparing the cultured enterococci criteria standard, 35 cfu with 130 cfu STV, and the HB 7,800 CN/100 mL criteria, roughly 65% of the exceedance days had EN exceeding 35 cfu when HB was not exceeding 7,800 CN/100 mL, and HB followed similar exceedances 62% of the time when EN did not exceed 35 cfu. However, the HIB are more specific for human fecal pollution sources likely to contain human pathogens, [36,63,64] and FIB are poorly correlated to HIB in urban rivers such as the Menomonee because FIB are derived from additional fecal pollution sources, which may not contain human pathogens [37]. Therefore, HIB may be a better proxy for high-risk fecal contamination events.

While these methods do provide estimates of risk from recreational water exposure, it is important to note that there are potential sources of uncertainty associated with these estimates [65]. Fecal indicator bacteria and human-associated bacteria markers are not perfect indicators of the collective presence of all pathogens and may underestimate the risks posed by pathogens. In addition, the relation between FIB and HB with pathogen presence in environmental waters and in sewage is variable, resulting in variable risk even with constant FIB and HB concentrations. The results presented in the current study are intended to be estimates that provide relative risk over different seasons and hydrologic conditions.

Previous monitoring and modeling with human-associated bacteria in the Great Lakes region, including the Menomonee River watershed, indicated that the incidence of human-specific pathogenic viruses increased with human-associated bacteria concentration (the sum of HB and another target marker in the *Lachnospiraceae* family, L2) [53]. The availability of continuous HIB estimates as developed in the current study could provide similar risk-based estimates of the likelihood of human pathogenic virus presence. With validation in additional water bodies, transfer of these concepts could prove useful in recreational waters and for evaluating the quality of drinking water sources before treatment.

HIB loads have previously been computed during defined monitoring periods on the Milwaukee River using discrete samples collected during baseflow and runoff events [36,37]. The current study built upon these techniques with regression models and optical sensors to provide loading estimates on a finer temporal scale (10-minute) and during time periods when discrete samples were not collected. These monitoring and modeling techniques have provided the means to estimate loads on variable time scales that are not limited to sample collection periods. For the current study, the flow-weighted proportion of sewage was multiplied by the sum of the daily volumes of water to provide an estimate of annual sewage contamination volume of 5.1 million m^3^ during the study period using sHM loadings. For individual HIB, there was not much difference in sewage contamination volume estimates using HB (4.9 million m^3^) as compared to L3 (5.5 million m^3^). Despite the consistency in results between HIB markers, including more than one measure of sewage influence is warranted given that each technique has associated uncertainties: these human-associated markers do have potential to cross react with fecal material from other animals even though they are thought to be primarily human-derived [38,66–68].

The use of surrogate models is not uncommon for predicting FIB in a more timely manner than the 18–24 hours that it takes for laboratory analysis. This has most commonly been done for prediction of FIB using models developed with explanatory variables chosen from web-available data (e.g. rainfall, cloud cover, wind speed and direction, currents), manually measured physical and water quality characteristics, or deployed water quality sensors (e.g. turbidity, β-D-glucuronidase) with telemetry at beaches [69–71] and in rivers [72–74]. These modeling efforts have focused on FIB prediction rather than human-associated markers. While FIB have been shown to be associated with gastrointestinal illness in recreational waters [75–77], they are not human specific and originate from many sources in the natural environment [78]. Attempts to correlate FIB with pathogen occurrence in natural waters have often been unsuccessful [53,79]. The current study included several FIB in modeling efforts but also shifted the focus from historical efforts to include human-associated bacteria that can be used to indicate contamination from human sewage. Previous work using results from laboratory analysis of water samples has demonstrated that the incidence of human pathogens increased with measured human-associated bacteria [53] and with modeled human-associated bacteria using modeling methods and field-deployed optical sensors as are used in the current study. This approach offers insight into concentrations of sewage contamination in a stream on a finer time scale and continuously over extended periods of time. Coupled with streamflow and load computation, monitoring systems deployed in different segments of a watershed can be used to determine areas with the greatest sewage loadings, enabling resource managers to focus remediation efforts in areas with the most concentrated sewage contamination.

Previous research for this monitoring location used two human-associated bacteria, three human-specific viruses, and the sum of the human-specific viruses (Sum of HSV) to compute the mean sewage contamination from all samples collected (low-flow and runoff-event periods) from April 2009 to March 2011 [5]. The estimated mean proportion of sewage for all samples collected (low-flow and runoff-events) varied between 0.0001 (0.01%) and 0.001 (0.1%) [5], as compared to 2.6 x 10^−7^ (0.000026%) and 0.028 (2.8%) estimated from Dec 2017 to Dec 2018 in the current study. Applying this approach to sewage content estimation in multiple areas of a watershed could help facilitate identification of areas and time periods with the greatest sewage contamination, enabling remediation projects to focus efforts. For example, consistent with previous research [5,36,37], loads and the proportion of sewage were greatest in the current study during event runoff periods and periods of active CSOs. Further, this information is important for receiving waters and can feed into water quality models that predict bacteria presence at beaches and other downstream recreational waters.

The current study established that the relation between optical properties and sewage was strengthened by including seasonality, flow conditions, and the nature of sewage contamination (CSO vs non-CSO periods) in the modeling structure (Table 2). Previous research has reported the use of currently available sensors that measure optical properties of water as surrogates to investigate raw and treated wastewater in multiple natural settings, including surface water, groundwater, and drinking water [18,28,80–83]. Optical properties of water, however, can be influenced by numerous factors other than wastewater, and can vary from watershed to watershed. Refining use of the optical signals by using HIB provided a direct indication of the presence and magnitude of human sewage waste. Inclusion of the seasonal term in each model as an interaction between DOM and FDOM provided a means to account for changing characteristics of DOM throughout the year. The background DOM in a watershed can vary with terrestrial and aquatic vegetation emergence and senescence throughout the year and as soil contact with runoff varies due to snow cover. The inclusion of flow and turbidity in these models also indicates that there is an association with physical characteristics and the current hydrologic condition in the watershed. Higher stream velocities can result in bacteria source changes along with the increased suspension of sediment particles. The regular inclusion of flow, and to a lesser extent turbidity, in selected models indicates that bacteria may be particle-associated, with some studies estimating approximately 40% of bacteria are attached to sediment, or that bacteria may behave similarly to other suspended matter [84,85]. The hydrologic conditions in the watershed have an impact on exfiltration from sanitary sewers with leakage of sewage increasing in many watersheds as soil saturation and flow increase [86]. Model cross-validation accuracy was generally improved for the non-CSO periods by separating samples collected during CSO discharge periods from those with no CSO influence. This result is likely due to the changing DOM composition as discharge from the combined sewer system delivers diluted sewage directly to the stream. Including separate models for CSO periods does present a complication for the application of this technique in other regions and for use in real-time estimation: for CSO events, separate CSO and non-CSO models will prompt corrections to bacteria estimations after the event has passed unless the stream monitoring and model prediction system is directly informed when CSO discharges occur, which is not common. However, the changing DOM composition can be followed in real-time with the use of optical sensors even though corrections to bacteria estimations post CSO event would be needed.

The monitoring and modeling system described in the current research indicates that HIB, FIB, and sewage contamination can be estimated with these methods, but there are limitations of the monitoring system and the models derived from resulting data that should be understood. For working models used for real-time predictions, initial discrete HIB and FIB measurements are necessary for model development and continuing discrete HIB and FIB measurements are necessary for model validation or refinement after the initial model development. In addition, it can be a challenge to represent the full range of HIB and FIB concentrations with discrete samples, and without representing the full range of variability, model extrapolation can have high levels of uncertainty. Therefore, it is judicious to limit the extent of extrapolation beyond the model calibration range. It has been demonstrated that models can be developed for a diverse variety of watersheds [53], but to transfer these methods to additional watersheds requires sufficient HIB or FIB measurements concurrent with optical property measurements because the relation between bacteria and DOM can change by watershed. Influences on DOM in streams such as seiche effects or highly diverse watershed influences from variable land use can influence the nature and quality of models [53]. As a result, in each watershed, there is a need for observations from all seasons and a representative variety of hydrologic conditions to account for the variable bacteria presence and DOM composition and magnitude to support development of these models.

In addition, it is important to understand that the variables used to estimate bacteria in this study are surrogates that are not direct measurements of bacteria presence. Models provided here are a statistical simplification of the relation between bacteria concentrations and the optical properties of water, turbidity, and season. These surrogate relationships may change over time based on changes in the watershed, and even without any changes in the watershed, the fit of each model has uncertainty associated with it. Sources of uncertainty may include climate and weather-related variability (e.g., differences between snowmelt and rainfall events, small-scale spatial variability of rainfall amount and intensity, air and water temperature), hydrologic variability (e.g., antecedent moisture conditions for runoff events, changing baseflow conditions), seasonal variability, anthropogenic activities (e.g., construction, repair of sanitary sewers), non-human fecal matter interferences (e.g., wildlife and domestic pet waste), and more [53,86–89]. Variables used in model development for the current study were chosen as an efficient way to estimate the collective variability from these sources with the understanding that it is not possible to represent all uncertainty. Even so, the results presented here do provide estimates of the relative magnitude of bacteria presence and associated sewage contamination under different environmental conditions that can be used by stakeholders to assist in watershed management decision making.

## Conclusions

Surrogate models developed in the current study provided a means to estimate bacteria concentrations over short temporal scales, incorporating variability driven by seasonal, diurnal, hydrologic, and other environmental changes. Recreational water quality criteria for primary contact designated uses were assessed for *E*. *coli*, enterococci, and HB, and regardless of the benchmark bacteria and concentration level chosen, the concentration exceedances were more prevalent during colder-weather months as opposed to warmer months with the exception being during runoff events, active CSO periods, and later in the summer. The monitoring and modeling system described in the current research indicates that HIB, FIB, and sewage contamination can be estimated with these methods, but there are limitations of the monitoring system and the models derived from resulting data that can be watershed specific. As a result, applying this technique elsewhere will require validation in each watershed to support development of these models. However, as shown in the current study, the application of optical sensors for estimation of HIB and FIB markers did provide a thorough assessment of bacterial presence, and human health risk in the Menomonee River.

## Supporting information

S1 Appendix Additional details on materials and methods and supplemental results figures and tables. (DOCX)
