# Supplementary material for: Modeled predictions of human-associated and fecal-indicator bacteria concentrations and loadings in the Menomonee River, Wisconsin using in-situ optical sensors
Source: PLoS One. 2023 Jun 8;18(6):e0286851. doi: 10.1371/journal.pone.0286851 (PMC10249839; doi:10.1371/journal.pone.0286851)
Supplement: S1 Appendix — (DOCX) [file pone.0286851.s001.docx]

# S1. Appendix. Additional details on materials and methods and supplemental results figures and tables.

## **Quality assurance sample collection**

Field blanks were collected by pumping approximately 1350 mL of deionized water through the 3-way valve and dispensing 350 mL into a 350 mL glass bottle and 1000 mL into a 1000 mL autoclaved polypropylene wedge-shaped bottle. Field blanks were analyzed for cultured-based markers, Enterococcus (EN, CFU/100 mL, colony-forming units per 100mL), *Escherichia coli* (*E. coli*, EC, CFU/100 mL) and fecal coliform (FC, CFU/100 mL), quantitative PCR assays, Human *Bacteroides* (HB, CN/100 mL, copy number per 100mL), Enterococcus (EN, CN/100 mL), *Escherichia coli* (*E. coli*, EC, CN/100 mL) and Human *Lachnospiraceae* (Lachno3, L3, CN/100 mL), dissolved organic carbon (DOC, mg/L) and fluorescence and absorbance properties (Table S1).

**Table S1. Field blank results for culture-based and Quantitative PCR Assays, dissolved organic carbon (DOC) and select laboratory absorbance and fluorescence properties.**

|  | **Result** | |
| --- | --- | --- |
|  | Sample 1 | Sample 2 |
| **Date/Time** | 05-16-18 / 10:30 | 09-11-18 / 12:20 |
| **Culture-Based Assays** |  |  |
| Enterococcus (EN, CFU/100 mL) | < 1.0 | 1.0 |
| *Escherichia coli* (*E. coli*, EC, CFU/100 mL) | < 1.0 | < 1.0 |
| Fecal Coliforms (FC, CFU/100 mL) | < 1.0 | 5.0 |
| **Quantitative PCR Assays** |  |  |
| Human *Bacteroides* (HB, CN/100 mL) | < 225 | < 225 |
| Enterococcus (EN, CN/100 mL) | < 225 | < 225 |
| *Escherichia coli* (*E. coli*, EC, CN/100 mL) | < 225 | < 225 |
| Human *Lachnospiraceae* (L3, CN/100 mL) | < 225 | < 225 |
| **Carbon Analysis** |  |  |
| Dissolved Organic Carbon (mg/L) | < 0.11 | < 0.11 |
| **Optical Analysis** |  |  |
| absorbance at 254 nm, (a254, AU) | < 0.006 | < 0.006 |
| S1.WS (Ex. 300-320 nm, Em. 430-474 nm) (RU) | < 0.015 | < 0.015 |
| S2.WS (Ex. 273-287 nm, Em. 430-474 nm) (RU) | < 0.015 | < 0.015 |
| S3.WS (Ex. 273-287 nm, Em. 330-370 nm) (RU) | < 0.034 | <0.034 |
| TUC (Ex. 265-385 nm, Em. 440-500 nm) (RU) | < 0.010 | < 0.010 |
| TUT (Ex. 275-285 nm, Em. 323-378 nm) (RU) | < 0.034 | < 0.034 |
| YFDOM (Ex. 360-370 nm, Em. 440-520 nm) (RU) | < 0.006 | < 0.006 |

[CFU/100 mL, colony forming unit per 100 milliliter; CN/100 mL, copy number per 100 mL; mg/L, milligrams per liter; nm, nanometers; AU, absorbance units; Ex., excitation; Em., Emission; RU, Raman units].

WRF sewage influent sample collection

Water Reclamation Facility (WRF) sewage influent samples are a 24-hour composite of manually collected grab samples over a five-day period. Initial sample collection began with a five-gallon Nalgene low density polyethylene (LDPE) carboy, from which an aliquot was taken and stored in a 2 L high density polyethylene (HDPE) bottle. Each subsequent day was sampled in the same manner as above. Each daily 2 L influent sample was stored in a refrigerator at 4 ⁰C. After collection of the last daily sample, an approximately 500 mL aliquot from each daily 2 L influent sample from the five-day period was poured into a 4 L polypropylene bottle and homogenized. A 1 L WRF influent sample was collected in a clean, autoclaved 1 L polypropylene bottle from this 4 L composite sample for fecal-indicator bacteria (FIB) and human-indicator bacteria (HIB) analysis. The 1 L WRF influent sample was then iced and shipped to the associated laboratory for FIB and HIB analysis. The 2 L bottles were clean, sterile bottles that were disposed of after each use. The five-gallon and 4 L bottles were cleaned (soap, tap, DI) after each five-day collection.

**
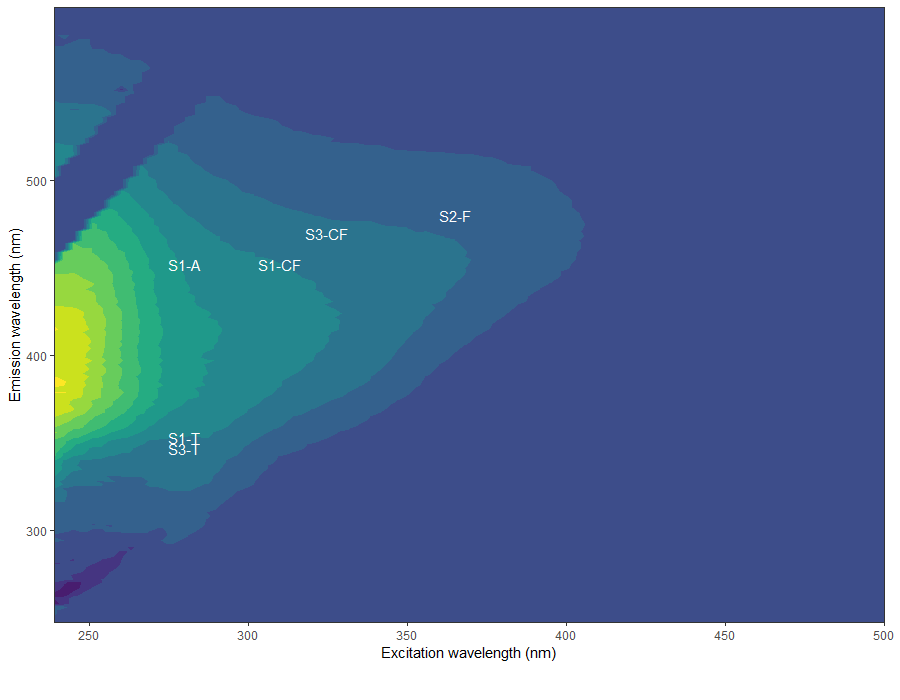
**

Fig S1. Example of an excitation-emission matrix (EEM) plot. The abbreviations indicate the location of the sensor’s excitation and emission wavelength pairs. Abbreviation’s excitation and emission wavelengths are defined in Table 1.

Optical sensors and the automated water sampler

Sensors were carefully cleaned manually every 1-4 weeks with lens paper and Q-tips, including sensors, manifold, and flow cells. The YSI sensor included a wiper that was cycled through a cleaning preceding each measurement. WET Labs and Turner sensor platforms lacked wipers, so were prone to varying degrees of biofouling between site visits. As part of the cleaning process, 18.2 (MΩ.cm) megohm-centimeter reagent water was pumped through the system to collect “dirty” measurements before cleaning. After the cleaning process, a second measurement was conducted using the same type of reagent water to obtain “clean” measurements (Table S2). The differences between the dirty and clean measurements were used to adjust readings for fouling.

**Table S2.** **Sensor readings with reagent water measured before (dirty) and after (clean) cleaning the sensors during site visits throughout study period.**

| **Date/Time** | **Measurement Name** | **YSI – Temperature (˚F)** | **YSI - Specific Conductance (µS/cm)** | **YSI – Turbidity (FNU)** | **S2-F (RFU)** | **S3-CF (mv)** | **S3-T (mv)** | **S1-CF (mv)** | **S1-A (mv)** | **S1-T (mv)** |
| --- | --- | --- | --- | --- | --- | --- | --- | --- | --- | --- |
| 11/7/2017 13:18 | Dirty | 19.9 | 5.4 | 0.9 | -0.89 | 41 | 277 | 177 | 21 | 54 |
| 11/7/2017 14:38 | Clean | 19.3 | 2.2 | 0.5 | -0.92 | 45 | 359 | 200 | 21 | 69 |
| 11/28/2017 9:38 | Dirty | 20.8 | 10.0 | 0.6 | -0.92 | 32 | 352 | 161 | 26 | 85 |
| 11/28/2017 12:17 | Clean | 20.4 | 3.6 | 0.2 | -0.62 | 38 | 426 | 166 | 21 | 52 |
| 12/15/2017 9:28 | Dirty | 21.4 | 9.7 | 0.3 | -0.6 | 45 | 427 | 156 | 32 | 90 |
| 12/15/2017 11:08 | Clean | 19.9 | 4.2 | 0.2 | -0.56 | 43 | 451 | 158 | 28 | 77 |
| 3/14/2018 14:58 | Dirty | 17.2 | 12.2 | 1.0 | -0.67 | 26 | 527 | 126 | 32 | 284 |
| 3/14/2018 16:38 | Clean | 17.5 | 4.9 | 0.5 | -0.63 | 45 | 376 | 132 | 9 | 311 |
| 5/16/2018 8:18 | Dirty | 22.9 | 18.2 | 0.8 | -0.59 | 7 | 565 | 156 | 10 | 558 |
| 5/16/2018 10:48 | Clean | 22.6 | 3.8 | 0.2 | -0.47 | 18 | 270 | 188 | 34 | 583 |
| 7/5/2018 10:08 | Dirty | 24.5 | 10.4 | 0.7 | -0.71 | 13 | 301 | 173 | 23 | 769 |
| 7/5/2018 12:58 | Clean | 25.5 | 1.6 | 0.4 | -0.65 | 20 | 386 | 187 | 24 | 748 |
| 8/7/2018 10:48 | Dirty | 23.0 | 7.8 | 1.3 | -0.72 | 14 | 378 | 202 | 18 | 852 |
| 8/7/2018 12:48 | Clean | 23.3 | 0.8 | 0.5 | -0.65 | 21 | 287 | 203 | 13 | 1553 |
| 9/11/2018 8:18 | Dirty | 23.4 | 13.5 | 1.0 | -0.69 | 7 | 302 | 195 | -7 | 507 |
| 9/11/2018 11:38 | Clean | 23.2 | 2.8 | 0.4 | -0.69 | 20 | 361 | 222 | 3 | 520 |
| 10/18/2018 9:38 | Dirty | 21.2 | 12.8 | 1.5 | -0.66 | 13 | 286 | 187 | 0 | 439 |
| 10/18/2018 11:08 | Clean | 20.8 | 3.3 | 0.6 | -0.65 | 20 | 297 | 200 | 2 | 462 |

Reagent water used was 18.2 (MΩ.cm) megohm-centimeter water. [°F, degree Fahrenheit; µS/cm, microsiemens per centimeter; FNU, formazin nephelometric unit; RFU, relative fluorescence units; mv, millivolts; S1-CF, S1-A, S1-T, S2-F, S3-CF, and S3-T are defined in Table S2].

Remote telemetry allowed unattended operation for initiating discrete sample collection and sensor measurements. Automated sampling was initiated, and real-time data was recorded using a Campbell Scientific CR 1000 data logger (Campbell Scientific, Logan, Utah) programmed for the control of the suite of sensors deployed, a variable-speed Masterflex I/P® peristaltic pump (Cole-Parmer, Vernon Hills, Illinois), 1/2” T-23 Electromni 3-way ball valve (Asahi/America, Malden, Massachusetts) and refrigerated automatic sampler (Teledyne ISCO, Lincoln, Nebraska). Ten-minute measurements were made on the eight-minute mark throughout the course of a day for the entire study period. The sensor measurement sequence proceeded as follows: at the six-minute mark the variable-speed peristaltic pump was set to reverse to purge water from the sample intake line, Menomonee River water was then pumped into the 3-way ball valve, through the peristaltic pump, into the WET Labs sensor, then the custom-designed manifold housing the Turner sensors, and into the YSI flow-through cell for one-minute and thirty seconds to flush any previous sample water out. After the continuous flush, the peristaltic pump stopped, the YSI EXO2 initiated a wiper sequence, and once complete the sensors made a measurement at the eight-minute mark throughout each hour. After the measurement, the pump turned back on and switched to reverse, purging the flow-through system of water (Fig 1). Norprene® I/P 73, Masterflex precision pump tubing (Cole-Parmer, Vernon Hills, Illinois) was used and was changed every 7 to 10 days throughout the study period. During periods of discrete flow-weighted sample collection, the datalogger was used to calculate flow rates and accumulated water volumes during low-flow and runoff event periods. After a predetermined volume was reached the 3-way valve would close to the flow-through sensor system and the automatic water sampler would pull water to its internal water detector, then switch directions and purge the sample line, prior to collection of 300 mL of sample water into a 300 mL glass bottle and a 1000 mL autoclaved polypropylene wedge bottle (Fig 1). Flow-weighted discrete samples were always collected on the two-minute mark after the sensor measurement sequence was complete.

Limit of Quantification

For all qPCR assays, the detection limit levels per 100 mL were dependent on the volume of water filtered from each sample. Water samples that amplify after 35 cycles were the low standard curve limit of 15 CN/reaction and were considered below limit of quantification (BLQ). BLQs were computed using equation 1

$$BLQ=(\left( \left( X \times Y \right)\div Z \right)\times100 (1)$$

Where *X* equals 15 CN per reaction divided by 5 µL of sample used in the reaction, *Y* equals 150 µL, the elution volume from DNA extraction, and *Z* equals the volume of water (mL) filtered from the sample. For example, if 100 mL of a water sample are filtered through a 0.22 µm cellulose esters filter then the BLQ would be

$$BLQ=(\left( \left( 15 CN\div5 \mu L) \times150 \mu L \right)\div100 mL \right)\times100$$

Thus, BLQ equals 450 CN/100 mL. Any sample that does not amplify before 40 cycles is reported as 0 CN/100 mL, a non-detection. The cultured plate count detection levels were 1 cfu/100 mL for Enterococcus, *Escherichia coli*, and fecal coliforms.

DNA extraction, qPCR assays, and culture-based analysis

The frozen filters were broken into small fragments using a sterile metal spatula. DNA was extracted using the MPBIO FastDNA® SPIN Kit for Soil (MP Biomedicals, Irvine, CA) according to manufacturer’s instructions, with the exception of the lysis step in which a bead beater (BioSpec, Bartlesville, OK) was used for 2 minutes and in the final step DNA was eluted using 150 µL of DNase/Pyrogen-Free Water (DES).

Quantitative PCR was carried out using an Applied Biosystems StepOne Plus™ Real-Time PCR System Thermal Cycling Block (Applied Biosystems; Foster City, CA) with Taqman hydrolysis probe chemistry. Previously published primers and probes were used for the human *Bacteroides* (HB) assay [1], with the exception that HF183F was used as the forward primer [2], the human *Lachnospiraceae* (L3) assay [3], the enterococci (EN) assay [4], and *Escherichia coli* (EC) assay (Table S3) [5]. Standard curves were generated based on 16 runs (in triplicate) and consisted of linearized plasmids containing the HB, L3, EN, and EC target sequences. The plasmids used for the standard curves are purified using a Qiagen mini plasmid prep kit (Qiagen, Valencia, CA) according to the manufacturer’s instructions. Standard curves were run with DNA serially diluted from 1.5 x 10^6^ to 1.5 x 10^1^ copies/reaction. No template controls were included in every run. For each run, two of the standard concentrations (as controls) and each sample was run in duplicate in a final volume of 25 µL with a final concentration of 1µM for each primer, 80 nM for the probe, 5 µL of sample DNA, and 12.5 µL of 2X Taqman® Gene Expression Master Mix Kit (Applied Biosystems; Foster City, CA). Amplification conditions consisted of the following cycles: 1 cycle at 50° C for 2 minutes to activate the uracil-N-glycosylase (UNG); 1 cycle at 95^o^ C for 10 minutes to inactivate the UNG and activate the Taq polymerase; 40 cycles of 95° C for 15 seconds; and 1 minute at 60^o^ C for HB, EC, and EN or 1 minute at 64^o^ for L3.

For qPCR values, a concentration of copy number (CN) per 100 mL of original sample was computed based on the volume of the sample used in the reaction. In most cases 200 mL were filtered for each sample (n=150), but for samples with low collection volume 100 mL were filtered (n=3). The limit of quantification was determined to be 15 copies per reaction, which corresponds to the last linear point on the standard curve. Depending on the volume filtered, 15 copies per reaction corresponds to 225 CN/100 mL for 200 mL filters or 450 CN/100 mL for 100 mL filters considering extracted DNA was eluted in a volume of 150 µL. Samples that showed positive amplification, but were below 15 copies in the reaction, were reported as below the limit of quantification (BLQ). All BLQ samples were given a censored concentration of 225 CN/100 mL (200 mL filtered) or 450 CN/100 mL (100 mL filtered).

Immediately after sample arrival at the laboratory, samples were analyzed for *Escherichia coli* (*E. coli*, EC), enterococci (EN), and fecal coliform (FC) bacteria using standard methods. Each sample was filtered through a 0.45-mm-pore-size nitrocellulose filter (0.47-mm diameter; Millipore, Billerica, MA) and filter was placed on differential media. For *E. coli* enumeration, filters were placed on modified mTEC agar, and incubated for 2 h at 35 ^0^C and the remaining 22 h at 44.5 ^0^C [6]. For EN enumeration, filters were placed on MEI agar and incubated for 24 h at 41 ^0^C [7]. For total FC enumeration, filters were placed on mFC agar and incubated for 24 h at 44.5 ^0^C [8]. After 24 h, plates were removed from the incubators and counted for colony forming units (CFU).

## Dissolved organic carbon and fluorescence and absorbance analysis

The non-purgeable organic carbon (NPOC) analysis method was employed using a Shimadzu TOC-V_CSH_ analyzer coupled with a Shimadzu ASI-V auto sampler. Water samples were sparged with 2 M HCL to remove all inorganic carbon prior to combustion. The resultant NPOC concentrations, given in units of mg C L^-1^, were calculated as a mean of three measurements with a maximum standard deviation of 0.1 mg C L^-1^. Based on a 1 mg C L^-1^ DOC standard (Organic Carbon standard, RICCA Chemical Company, Arlington, Texas) measured from 2017 to 2018, a method detection limit (MDL) of 0.108 mg C L^-1^ was computed based on the student t-value for 95% confidence and 59 observations multiplied by the standard deviation of the 1 mg C L^-1^ DOC standard blank corrected results.

Fluorescence excitation-emission matrix (EEM) and absorbance scan measurements were performed using a Jobin Yvon Aqualog benchtop Spectrofluorometer (HORIBA Scientific, Piscataway, New Jersey). Absorbance and fluorescence analysis were performed in a 1-cm path-length quartz cuvette. Samples were diluted with ultra-pure 18.2 megohm water and re-run if UV-Visible absorption data at 254 nm (A_254_) exceeded 0.3 absorbance units to limit inner filter effect problems [9–12]. Absorbance and fluorescence spectra were corrected for inner filtering effects, Rayleigh 1^st^ and 2^nd^ order masking using automated algorithms supplied with the Aqualog software (HORIBA Scientific, Piscataway, New Jersey). All absorbance and fluorescence sample results were blank corrected and fluorescence intensity was normalized to Raman units (RU) using a Starna (Starna Cells, Inc., RM-H2O, California) certified Raman Water Fluorescence Reference standard.

**Table S3. Primers and Probes for Human *Bacteroides* (HB)*,* Human *Lachnospiraceae* 3 (L3), *E. Coli* (EC), *Enterococcus faecalis* (EN), and salmon sperm DNA.**

| **Target** | **Primer/Probe** | **Sequence** | **Amplicon Size, base pair (bp)** | **Reference** |
| --- | --- | --- | --- | --- |
| Human *Bacteroides* (HB) | HF183F  HF241R  HF193p | 5′ATC ATG AGT TCA CAT GTC CG3’  5’CGT TAC CCC GCC TAC TAT CTA ATG3’  5’[6FAM]-TCC GGT AGA CGA TGG GGA TGC GTT [MGB-NFQ] 3’ | 86 bp | [1,2] |
| Human *Lachnospiraceae* 3 (L3) | Lachno3-F  Lachno3-R  Lachnop | 5’CAA CGC GAA GAA CCT TAC CAA A3’  5’CCC AGA GTG CCC ACC TTA AAT3’  5’[6FAM]-CTC TGA CCG GTC TTT AAT CGG A [MGB-NFQ] 3’ | 187 bp | [3] |
| *E. coli* (EC) | uidA1663F  uidA1790R  uidA1729p | 5’GCG ACC TCG CAA GGC ATA3’  5’GAT TCA TTG TTT GCC TCC CTG CTG CG3’  5’[6FAM]-TGCAGCAGAAAAGCCGCCGACTTCGG [MGB-NFQ] 3’ | 127 bp | [5] |
| *Enterococcus faecalis* (EN)  23s rDNA  ATCC29212 | Entero1F-G  Entero2R  Enterop | 5’GAG AAA TTC CAA ACG AAC TTG3’  5’CAG TGC TCT ACC TCC ATC ATT3’  5’[6FAM]-TGGTTCTCTCCGAAATAGCTTTAGGGCTA[ MGB-NFQ] 3’ | 92 bp | [4] |
| Salmon sperm DNA | Sketa2F  Sketa2R  Sketap | 5’GGT TTC CGC AGC TGG G3’  5’CCG AGC CGT CCT GGT C3’  5’[FAM]- AGT CGC AGG CGG CCA CCG T [MGB-NFQ]3’ | 77 bp | [4] |

[bp, base pair].

## U.S. Geological Survey National Water Information System Data Retrieval

Streamflow and the uncorrected time-series sensor data from the Menomonee River at 16^th^ Street at Milwaukee, Wisconsin (MRM, 04087142) are archived in the U.S. Geological Survey National Water Information System (NWIS, Table S4) [13]. Human-associated indicator bacteria (HIB), fecal indicator bacteria (FIB), and dissolved organic carbon (DOC) from the MRM sampling location are archived in the U.S. Geological Survey NWIS and can be accessed using the following URL, <https://nwis.waterdata.usgs.gov/wi/nwis/qwdata?search_criteria=search_site_no&submitted_form=introduction>. Using the above URL, enter the MRM station number from Table S4, the time period of study (11/01/2017 – 12/31/2018), and the U.S. Geological Survey parameter codes in Table S5 to retrieve water quality data from the MRM location for the duration of the study [14].

**Table S4. U.S. Geological Survey Streamflow location name, station ID, abbreviation, and URL for accessing streamflow and time-series sensor data.**

| **USGS streamflow location name** | **USGS station number** | **Streamflow location abbreviation** | **URL** |
| --- | --- | --- | --- |
| Menomonee River at 16^th^ Street at Milwaukee, WI | 04087142 | MRM | <https://nwis.waterdata.usgs.gov/wi/nwis/uv?cb_00010=on&cb_00060=on&cb_32322=on&cb_63680=on&cb_75971=on&cb_75971=on&cb_75971=on&cb_75971=on&cb_75971=on&format=gif_default&site_no=04087142&period=&begin_date=2017-11-01&end_date=2018-12-31> |

**Table S5. U.S. Geological Survey parameter codes and names for fecal indicator bacteria, human-associated indicator bacteria, and dissolved organic carbon.**

| **U.S. Geological Survey parameter code** | **U.S. Geological Survey parameter name** |
| --- | --- |
| 31616 | Fecal coliforms, M-FC MF (0.45 micron) method, water, colony forming units per 100 milliliters |
| 90909 | Enterococci, mEI MF method, water, colony forming units per 100 ml |
| 31633 | *Escherichia coli*, m-TEC MF method, water, colony forming units per 100 milliliters |
| 31896 | *Escherichia coli* uidA gene, bacteria, water, filtered (0.22 micron filter), quantitative polymerase chain reaction (qPCR) method, copy numbers per 100 milliliters |
| 31745 | Enterococci, water, quantitative polymerase chain reaction (qPCR) method, copy numbers per 100 milliliters (cn/100 mL) |
| 31742 | Human *Bacteroides* bacteria, water, quantitative polymerase chain reaction (qPCR) method, copy numbers per 100 milliliters (cn/100 mL) |
| 31895 | Human *Lachnospiraceae* 3 bacteria Lachno3 gene, water, filtered (0.22 micron filter), quantitative polymerase chain reaction (qPCR) method, copy numbers per 100 milliliters |
| 00681 | Organic carbon, water, filtered, milligrams per liter |

**Table S6. Defined start and end periods for Menomonee River Combined Sewer Overflows (CSOs) that occurred upstream of the Menomonee River at North Emmber lane, Milwaukee, Wisconsin.**

| **CSO Start Date/Time** | **CSO End Date/Time** | **Number of Samples collected** |
| --- | --- | --- |
| June 18, 2018 / 14:00 | June 19, 2018 / 13:00 | 6 |
| August 20, 2018 / 22:00 | August 21, 2018 / 7:00 | 7 |
| August 27, 2018 / 1:00 | August 27, 2018 / 7:00 | 0 |
| August 28, 2018 / 3:00 | August 29, 2018 / 7:00 | 0 |
| September 5, 2018 / 17:00 | September 6, 2018 7:00 | 7 |
| October 1, 2018 / 20:00 | October 2, 2018 / 14:00 | 6 |

Number of grab surface water stream samples collected at the Menomonee River at 16^th^ St. location between the CSO start and end date/time. [CSO, combined sewer overflow].

**Table S7. Low-flow, event-runoff, and event-combined sewer overflow (CSO) period mean, geometric mean (geomean), median, maximum concentrations, occurrence (percent %) of bacteria markers, and number of samples (n) for all water samples collected from the Menomonee River at 16^th^ St. at Milwaukee, Wisconsin, December 2017 to December 2018.**

|  |  | **Concentration (CFU/100 mL, culture;**  **CN/100 mL, qPCR)** | | | |  |  |
| --- | --- | --- | --- | --- | --- | --- | --- |
| **Flow Condition** | **Bacteria** | **Mean** | **Geomean** | **Median** | **Maximum** | **Occurrence (%)** | **Number of samples collected (n)** |
| Low-flow | Fecal coliform (FC, culture) | 312 | 38 | 265 | 830 | 92 | 24 |
|  | Enterococci (EN, culture) | 52 | 8 | 10 | 490 | 79 | 28 |
|  | Enterococci (EN, qPCR) | 12,447 | 5,632 | 3,661 | 55,921 | 100 | 34 |
|  | *E. coli* (EC, culture) | 51 | 23 | 26 | 289 | 88 | 34 |
|  | *E. coli* (EC, qPCR) | 128 | 7 | 0 | 549 | 32 | 34 |
|  | HB (qPCR) | 1,807 | 712 | 1,301 | 6,664 | 94 | 34 |
|  | L3 (qPCR) | 1,338 | 112 | 833 | 6,090 | 65 | 34 |
|  | sHM (qPCR) | 3,145 | 1,022 | 2,168 | 12,754 | 94 | 34 |
| Event-Runoff | Fecal coliform (FC, culture) | 8,888 | 1,281 | 3,500 | 93,000 | 100 | 68 |
|  | Enterococci (EN, culture) | 2,335 | 216 | 350 | 14,700 | 94 | 68 |
|  | Enterococci (EN, qPCR) | 108,714 | 29,972 | 21,064 | 1,211,645 | 100 | 76 |
|  | *E. coli* (EC, culture) | 1,359 | 321 | 410 | 8,800 | 100 | 76 |
|  | *E. coli* (EC, qPCR) | 2,073 | 114 | 446 | 12,854 | 63 | 76 |
|  | HB (qPCR) | 7,157 | 763 | 2,713 | 50,612 | 83 | 76 |
|  | L3 (qPCR) | 6,654 | 469 | 2,393 | 61,738 | 75 | 76 |
|  | sHM (qPCR) | 13,810 | 1,361 | 5,354 | 112,350 | 84 | 76 |
| Event-CSO | Fecal coliform (FC, culture) | 273,909 | 60,062 | 51,000 | 3,720,000 | 100 | 43 |
|  | Enterococci (EN, culture) | 7,787 | 3,840 | 3,200 | 32,400 | 100 | 43 |
|  | Enterococci (EN, qPCR) | 600,406 | 321,288 | 391,817 | 3,649,422 | 100 | 43 |
|  | *E. coli* (EC, culture) | 13,002 | 6,793 | 8,100 | 73,000 | 100 | 43 |
|  | *E. coli* (EC, qPCR) | 19,856 | 8,756 | 11,997 | 85,464 | 98 | 43 |
|  | HB (qPCR) | 159,505 | 17,147 | 21,257 | 1,268,921 | 95 | 43 |
|  | L3 (qPCR) | 144,776 | 27,377 | 40,365 | 738,415 | 98 | 43 |
|  | sHM (qPCR) | 304,281 | 49,543 | 92,913 | 2,007,336 | 98 | 43 |

[sHM sum of indicator markers (HB + L3); L3, human *Lachnospiraceae* 3; HB, human *Bacteroides*; culture, CFU, colony forming units; qPCR, CN, copy number; mL, milliliter].

**Table S8. Water reclamation facility (WRF) influent mean, geometric mean (geomean), median, maximum concentrations (CN/100 mL), occurrence (percent %) of bacteria markers, and number of samples (n) for all water samples collected from Jones Island WRF, Milwaukee, Wisconsin, November 2017 to December 2018.**

| **Bacteria Markers** | **Mean (CN/100 mL)** | **Geomean (CN/100 mL)** | **Median (CN/100 mL)** | **Maximum (CN/100 mL)** | **Occurrence**  **(%)** | **Number of samples collected (n)** |
| --- | --- | --- | --- | --- | --- | --- |
| HB | 2.79E+07 | 2.05E+07 | 3.00E+07 | 5.17E+07 | 100 | 12 |
| Enterococci (EN) | 7.54E+08 | 6.96E+08 | 7.88E+08 | 1.33E+09 | 100 | 12 |
| *E. Coli* (EC) | 1.24E+06 | 1.00E+06 | 9.06E+05 | 4.86E+06 | 100 | 12 |
| L3 | 2.68E+07 | 1.57E+07 | 2.69E+07 | 4.49E+07 | 100 | 12 |
| sHM | 5.46E+07 | 3.76E+07 | 5.70E+07 | 9.66E+07 | 100 | 12 |

[sHM, sum of human-indicator markers; L3, human *Lachnospiraceae* 3; HB, human *Bacteroides*; CN, copy number; mL, milliliter].


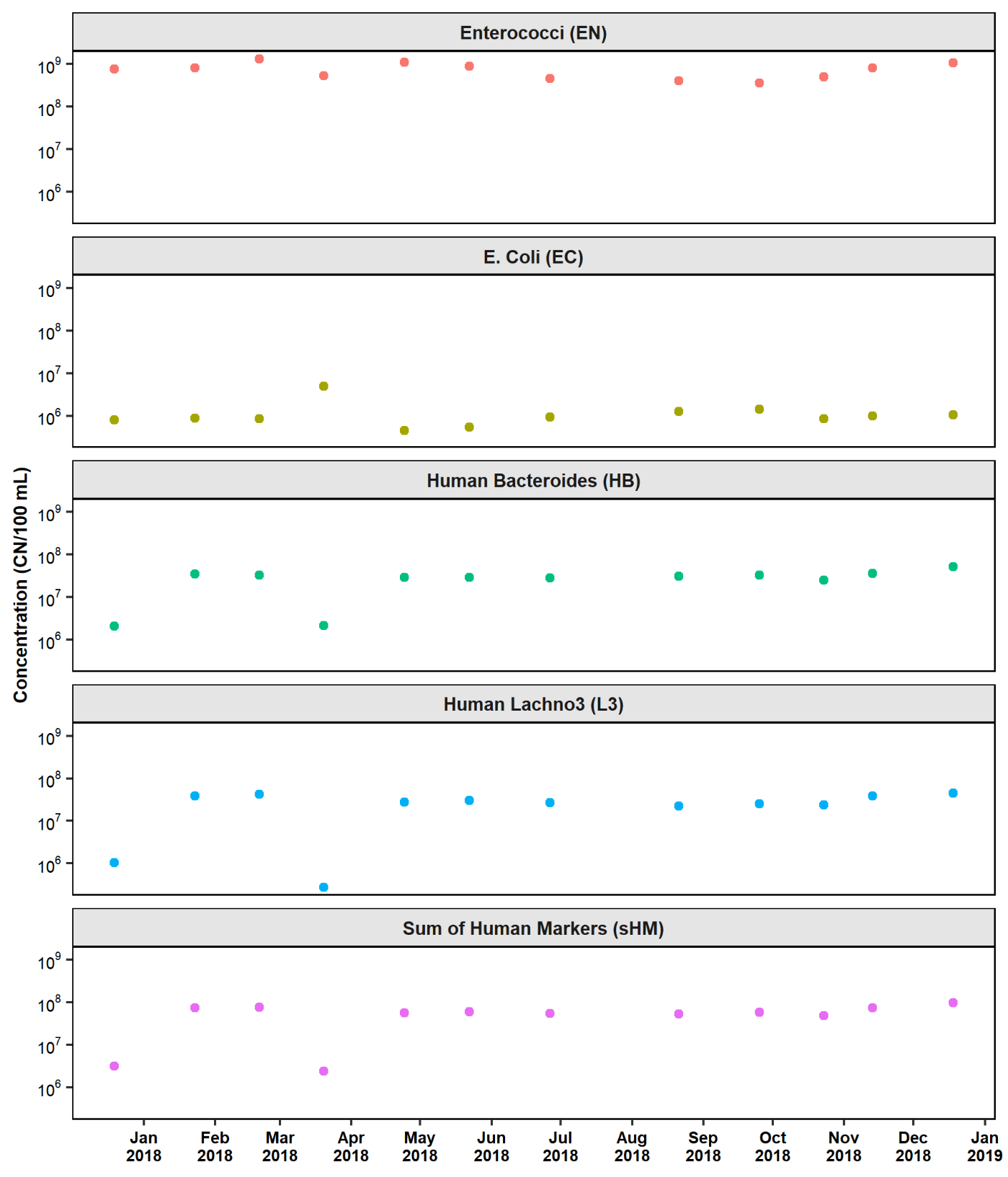


**Fig S2. Time series of concentrations in water reclamation facility (WRF) influent collected from Jones Island WRF in Milwaukee, WI, November 2017 to December 2018.** [CN, copy numbers; mL, milliliter].


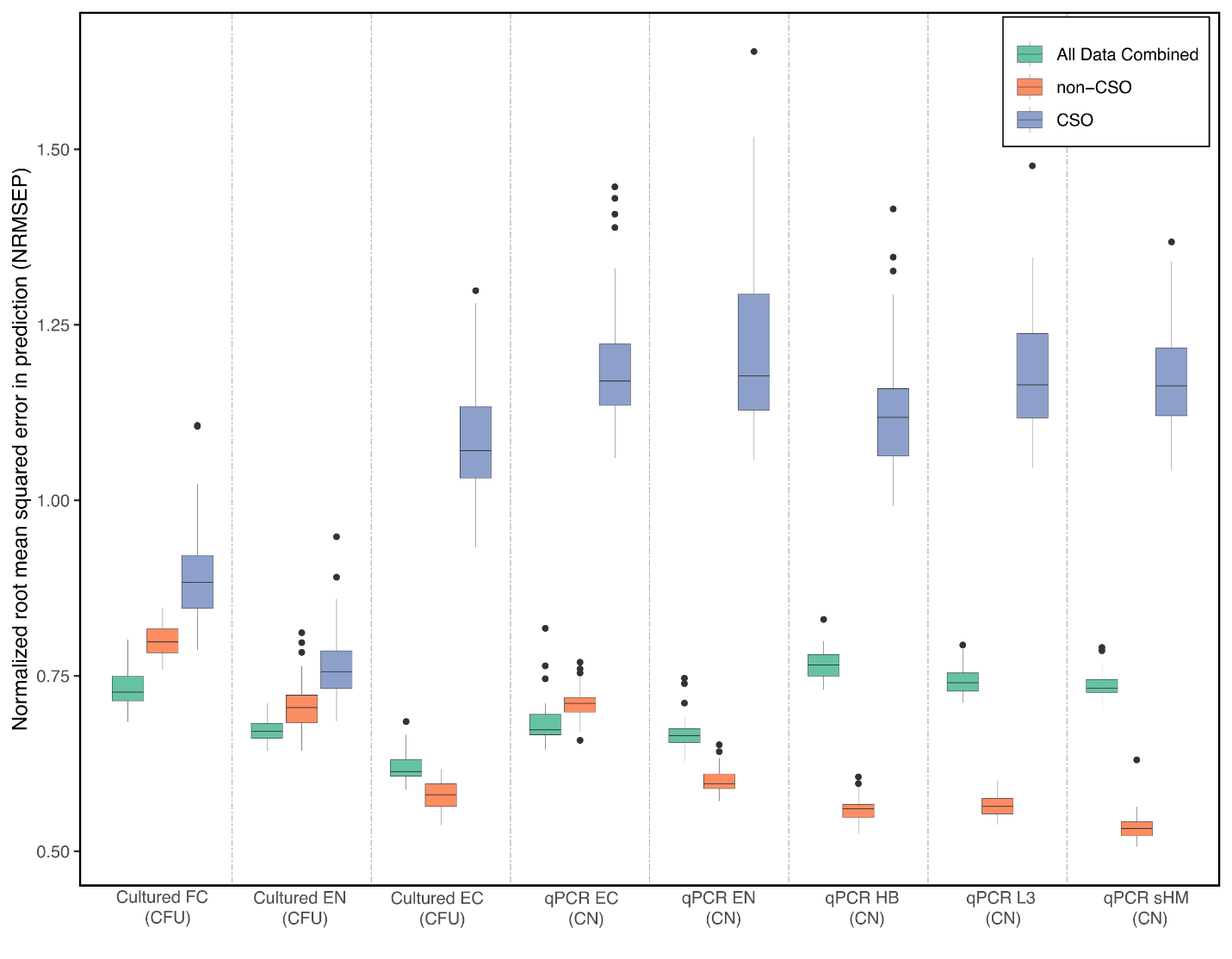


**Fig S3. Boxplots of cross-validation error as normalized root mean square error in prediction (NRMSEP) reported in log base 10 of the response variable for 50 replications using all data combined, non-combined sewer overflow (non-CSO), and CSO models.** The grey dashed line separates the three box plots by bacteria. [Boxes, 25th to 75th percentiles; horizontal line, median; whiskers, data within 1.5× the interquartile range (IQR); and circles, values outside 1.5× the IQR. [FC, fecal coliform; EN, enterococci; EC, *Escherichia coli*; HB, human *Bacteroides*; L3, human *Lachnospiraceae*; sHM, sum of human markers (HB + L3); CFU, colony forming units per 100 milliliter; CN, copy numbers per 100 milliliter].

**Table S9. Coefficients of explanatory variables for regression models to estimate bacteria concentrations during combined sewer overflow (CSO) periods, non-CSO periods, and all data combined periods (combo).** NS, variable not selected in model; Seasonal sine or cosine, sine or cosine of (Julian day/number of days in year)2π; interaction terms are designated by column headers that include a colon separating the two interacting independent variables.

| **Response** | **Model** | **MRM Mean one-hour flow** | **Turbidity** | **Seasonal sine** | **Seasonal cosine** | **S1-CF** | | **S1-A** | **S2-F** | **Seasonal sine: MRM Mean one-hour flow** | | **Seasonal cosine: MRM Mean one-hour flow** | |
| --- | --- | --- | --- | --- | --- | --- | --- | --- | --- | --- | --- | --- | --- |
| Cultured FC (CFU) | CSO | NS | NS | 1.71 | -0.493 | NS | | -0.463 | -1.04 | NS | | NS | |
| Cultured FC (CFU) | non-CSO | 0.0293 | NS | -1.11 | -1.33 | NS | | 0.222 | 0.246 | 0.0106 | | 0.0225 | |
| Cultured FC (CFU) | combo | 0.0281 | NS | 1.09 | 0.98 | -0.645 | | 0.0745 | 0.571 | 0.00048 | | 0.0202 | |
| Cultured EN (CFU) | CSO | NS | NS | 1.06 | 0.269 | -1.36 | | NS | -1.06 | NS | | NS | |
| Cultured EN (CFU) | non-CSO | 0.0396 | 0.00207 | -1.09 | 0.564 | 3.61 | | -2.19 | -1.64 | NS | | NS | |
| Cultured EN (CFU) | combo | 0.0449 | NS | -1.11 | 0.00593 | 3.67 | | -2.2 | -1.75 | 0.03 | | 0.0162 | |
| Cultured EC (CFU) | CSO | 0.0378 | NS | -1.6 | -0.347 | NS | | -0.125 | 0.231 | 0.0141 | | 0.0221 | |
| Cultured EC (CFU) | non-CSO | 0.0408 | -0.00152 | -1.1 | 0.806 | 0.946 | | -0.771 | -0.233 | NS | | NS | |
| Cultured EC (CFU) | combo | 0.038 | NS | -0.826 | 0.429 | 1.03 | | -0.829 | -0.336 | 0.014 | | 0.0192 | |
| qPCR EC (CN) | CSO | NS | NS | 0.516 | -1.69 | NS | | 0.128 | -1.56 | NS | | NS | |
| qPCR EC (CN) | non-CSO | 0.0368 | 0.00045 | -3.5 | 0.671 | NS | | 0.792 | 0.112 | NS | | NS | |
| qPCR EC (CN) | combo | 0.037 | NS | -2.29 | 0.846 | -0.586 | | 0.911 | 0.321 | 0.0184 | | 0.00813 | |
| qPCR EN (CN) | CSO | NS | NS | 1.05 | -1.62 | NS | | -0.0166 | -1.74 | NS | | NS | |
| qPCR EN (CN) | non-CSO | 0.0281 | -0.000919 | -1.35 | -0.0968 | 0.243 | | NS | -0.772 | NS | | NS | |
| qPCR EN (CN) | combo | 0.0296 | NS | -0.28 | 0.994 | -1.36 | | 0.837 | 0.242 | 0.00971 | | 0.00634 | |
| qPCR HB (CN) | CSO | NS | NS | 0.688 | -0.685 | NS | | 0.771 | -2.43 | NS | | NS | |
| qPCR HB (CN) | non-CSO | NS | 0.00939 | -1.15 | 1.07 | NS | | 1.29 | -1.4 | NS | | NS | |
| qPCR HB (CN) | combo | 0.0275 | NS | -0.497 | 3.96 | -2.27 | | 2.35 | 0.618 | 0.00585 | | -0.0187 | |
| qPCR L3 (CN) | CSO | NS | NS | 1.22 | -1.8 | NS | | 0.508 | -2.52 | NS | | NS | |
| qPCR L3 (CN) | non-CSO | 0.0305 | NS | -2.03 | 1.09 | NS | | 1.26 | -1.1 | 0.0219 | | -0.0193 | |
| qPCR L3 (CN) | combo | 0.0295 | NS | -0.133 | 2.93 | -2.21 | | 2.11 | 0.373 | 0.0102 | | -0.0177 | |
| qPCR SHM (CN) | CSO | NS | NS | 1.17 | -1.45 | NS | | 0.572 | -2.51 | NS | | NS | |
| qPCR SHM (CN) | non-CSO | 0.0286 | NS | -1.81 | 1.38 | NS | | 1.26 | -1.12 | 0.0192 | | -0.0202 | |
| qPCR SHM (CN) | combo | 0.0268 | NS | 0.0512 | 3.07 | -2.17 | | 2.12 | 0.292 | 0.00753 | | -0.0187 | |
| **Response** | **Model** | **Seasonal sine: S1-CF** | **Seasonal cosine: S1-CF** | **Seasonal sine: S1-A** | **Seasonal cosine: S1-A** | | **Seasonal sine: S2-F** | | **Seasonal cosine: S2-F** | | **Seasonal sine: Turbidity** | **Seasonal cosine: Turbidity** |  |
| Cultured FC (CFU) | CSO | NS | NS | NS | NS | | 2.05 | | -1.07 | | NS | NS |  |
| Cultured FC (CFU) | non-CSO | NS | NS | -0.62 | 0.456 | | 1.95 | | -0.0418 | | NS | NS |  |
| Cultured FC (CFU) | combo | 3.67 | 3.64 | -2.85 | -3.18 | | -1.59 | | -1.07 | | NS | NS |  |
| Cultured EN (CFU) | CSO | NS | NS | NS | NS | | 1.95 | | 1.23 | | NS | NS |  |
| Cultured EN (CFU) | non-CSO | 2.81 | 4.83 | -2.44 | -3.33 | | 0.894 | | -1.01 | | 0.00851 | -0.00828 |  |
| Cultured EN (CFU) | combo | 2.82 | 4.23 | -2.4 | -2.81 | | 0.773 | | -0.621 | | NS | NS |  |
| Cultured EC (CFU) | CSO | NS | NS | 0.0274 | -0.885 | | 0.791 | | 1.95 | | NS | NS |  |
| Cultured EC (CFU) | non-CSO | 2.45 | 2.37 | -1.43 | -2.72 | | -0.698 | | 0.75 | | 0.00954 | -0.0111 |  |
| Cultured EC (CFU) | combo | 2.61 | 1.9 | -1.64 | -2.29 | | -0.792 | | 1.02 | | NS | NS |  |
| qPCR EC (CN) | CSO | NS | NS | NS | NS | | 1.57 | | 0.356 | | NS | NS |  |
| qPCR EC (CN) | non-CSO | NS | NS | 0.704 | -0.296 | | 1.51 | | 0.15 | | 0.0104 | -0.00888 |  |
| qPCR EC (CN) | combo | 0.503 | 1.14 | 0.169 | -1.38 | | 0.645 | | 0.325 | | NS | NS |  |
| qPCR EN (CN) | CSO | NS | NS | NS | NS | | 0.8 | | 0.297 | | NS | NS |  |
| qPCR EN (CN) | non-CSO | 0.366 | 0.187 | NS | NS | | 0.161 | | -0.0116 | | 0.00684 | -0.00272 |  |
| qPCR EN (CN) | combo | 0.717 | 1.64 | -0.318 | -1.97 | | -0.641 | | 0.609 | | NS | NS |  |
| qPCR HB (CN) | CSO | NS | NS | NS | NS | | 0.507 | | 0.443 | | NS | NS |  |
| qPCR HB (CN) | non-CSO | NS | NS | -0.119 | 0.145 | | 1.3 | | -0.198 | | 0.0103 | -0.0128 |  |
| qPCR HB (CN) | combo | 0.35 | 4.21 | 0.535 | -4.46 | | -1.14 | | -0.292 | | NS | NS |  |
| qPCR L3 (CN) | CSO | NS | NS | NS | NS | | 0.749 | | 0.33 | | NS | NS |  |
| qPCR L3 (CN) | non-CSO | NS | NS | 0.545 | 0.235 | | 0.515 | | -0.421 | | NS | NS |  |
| qPCR L3 (CN) | combo | 0.915 | 4.91 | -0.146 | -4.29 | | -1.19 | | -0.791 | | NS | NS |  |
| qPCR SHM (CN) | CSO | NS | NS | NS | NS | | 0.652 | | 0.358 | | NS | NS |  |
| qPCR SHM (CN) | non-CSO | NS | NS | 0.547 | 0.0595 | | 0.349 | | -0.321 | | NS | NS |  |
| qPCR SHM (CN) | combo | 0.448 | 5.21 | 0.0762 | -4.52 | | -0.996 | | -0.862 | | NS | NS |  |

[FC, fecal coliform; EN, enterococci; EC, *E. coli*; HB, human *Bacteroides*; L3, human *Lachnospiraceae*; sHM, sum of human markers (HB+L3)].


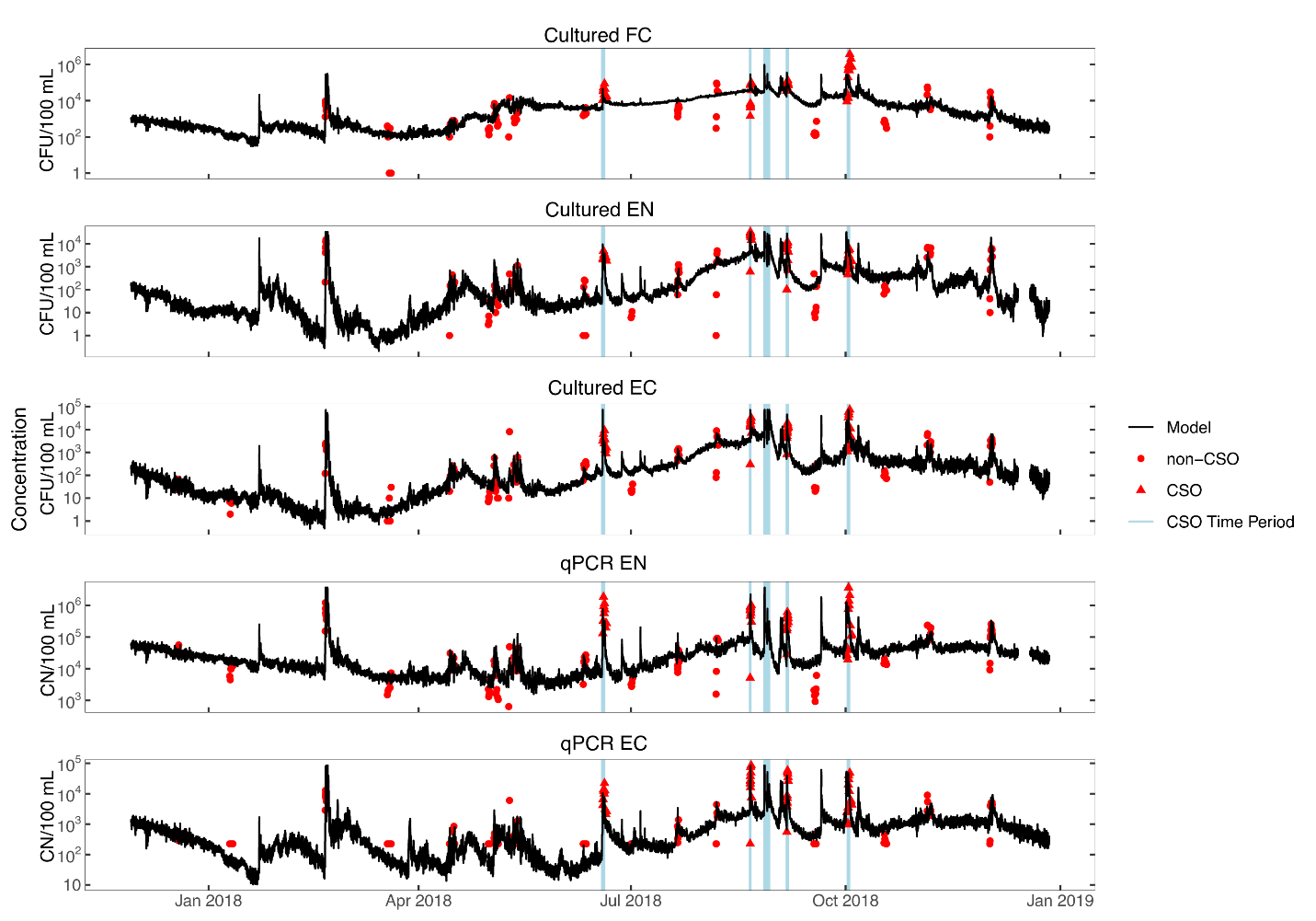


**Fig S4. Stream sample results and predictions of continuous cultured and qPCR fecal-indicator bacteria concentrations at 10-minute intervals for the Menomonee River in Milwaukee, Wisconsin, December** **2017 to December 2018**. Vertical, blue-shaded regions, represent event-CSO time periods. Predictions for all time periods were derived using the “CSO model” for CSO periods and the “non-CSO model” for non-CSO time periods. When estimated values exceed the stream sample concentration data from the model calibration, the estimations are designated as EN > 3.2 x 10^4^ CFU/100 mL, EC > 7.3 x 10^4^ CFU/100 mL, FC > 3.7 x 10^6^ CFU/100 mL, EN > 3.6 x 10^6^ CN/100 mL, and EC > 8.5 x 10^4^ CN/100 mL. [CFU/100 mL, colony forming units per 100 milliliter; CN/100 mL, copy number per 100 milliliter; qPCR, quantitative polymerase chain reaction; FC, fecal coliforms; EN, enterococci; EC, *E.* *Coli*].


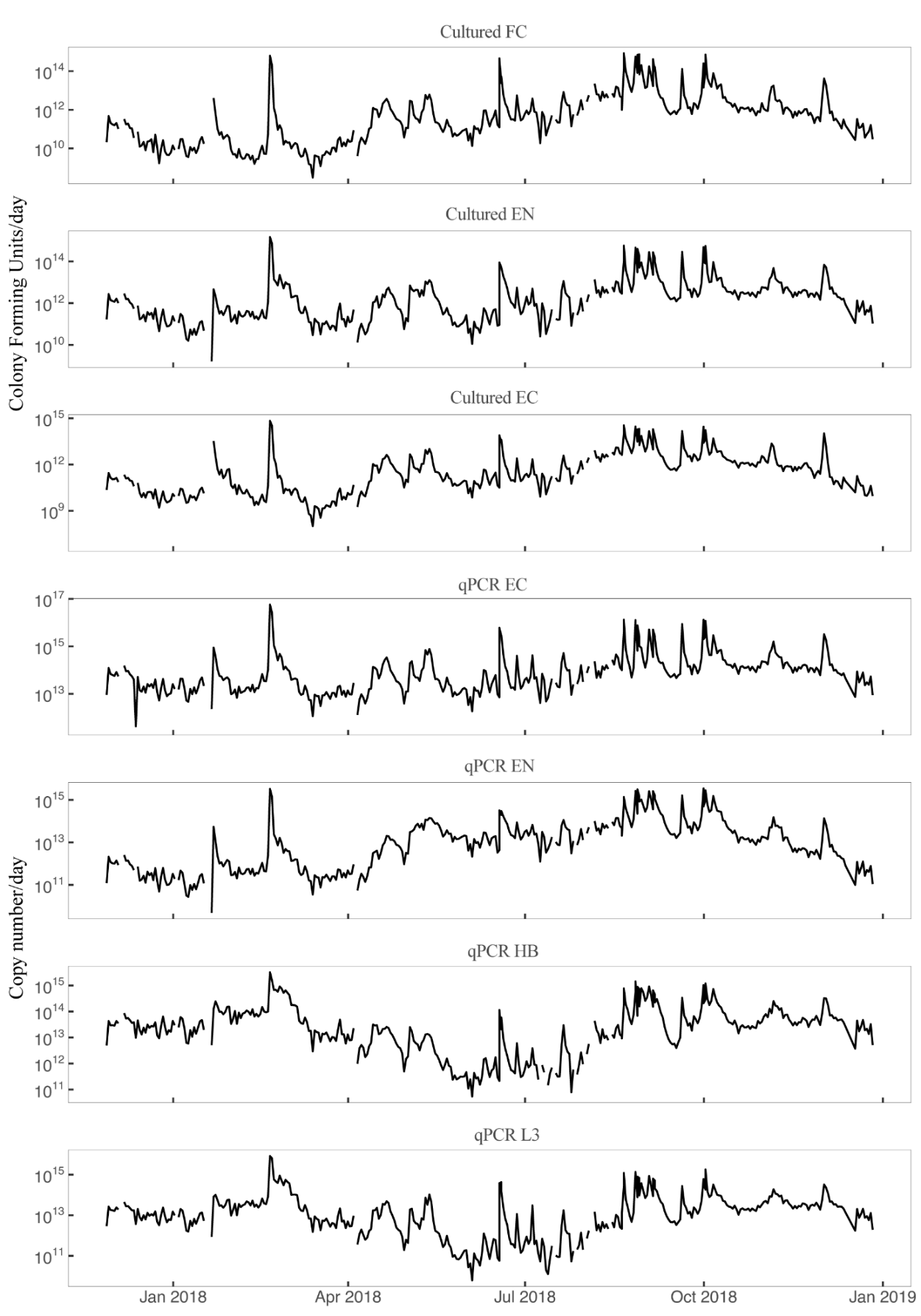


**Fig S5. Estimates of daily human-associated and fecal-indicator bacteria loads (black line) for the Menomonee River in Milwaukee, Wisconsin, December 2017-Decemeber 2018.** [ qPCR, quantitative polymerase chain reaction; FC, fecal coliforms; EN, enterococci; EC, *E.* *Coli*; HB, human *Bacteroides*; L3, human *Lachnospiraceae*].

# Disclaimer

Any use of trade, product, or firm names is for descriptive purposes only and does not imply endorsement by the U.S. Government.

# References

1. Kildare BJ, Leutenegger CM, McSwain BS, Bambic DG, Rajal VB, Wuertz S. 16S rRNA-based assays for quantitative detection of universal, human-, cow-, and dog-specific fecal Bacteroidales: A Bayesian approach. Water Research. 2007;41: 3701–3715. doi:10.1016/j.watres.2007.06.037

2. Bernhard AE, Field KG. A PCR Assay To Discriminate Human and Ruminant Feces on the Basis of Host Differences in Bacteroides-Prevotella Genes Encoding 16S rRNA. Appl Environ Microbiol. 2000;66: 4571–4574. doi:10.1128/AEM.66.10.4571-4574.2000

3. Feng S, Bootsma M, McLellan SL. Human-Associated Lachnospiraceae Genetic Markers Improve Detection of Fecal Pollution Sources in Urban Waters. Appl Environ Microbiol. 2018;84: e00309-18. doi:10.1128/AEM.00309-18

4. U.S. Environmental Protection Agency. Method 1611: Enterococci in Water by TaqMan®Quantitative Polymerase Chain Reaction (qPCR) Assay. Washington, D.C.: U.S. Environmental Protection Agency Office of Water; 2012 Oct p. 35. Report No.: EPA-821-R-12-008. Available: http://water.epa.gov/scitech/methods/cwa/bioindicators/upload/Method-1611-Enterococci-in-Water-by-TaqMan-Quantitative-Polymerase-Chain-Reaction-qPCR-Assay.pdf

5. Sauer EP, VandeWalle JL, Bootsma MJ, McLellan SL. Detection of the human specific Bacteroides genetic marker provides evidence of widespread sewage contamination of stormwater in the urban environment. Water Research. 2011;45: 4081–4091. doi:10.1016/j.watres.2011.04.049

6. U.S. Environmental Protection Agency. Method 1603: Escherichia coli (E. coli) in water by membrane filtration using modified membrane-thermotolerant Escherichia coli agar (modified mTEC). US Environmental Protection Agency, Washington [DC]; 2002. Report No.: EPA 821-R-02-023.

7. U.S. Environmental Protection Agency. Method 1600: Enterococci in water by membrane filtration using membrane-Enterococcus indoxyl-B-d-glucoside agar (mEI). US Environmental Protection Agency Washington, DC; 2006. Report No.: EPA-821-R-06-009.

8. American Public Health Association. Standard Methods for the Examination of Water and Wastewater. twentieth. Washington, D.C.: American Public Health Association.; 1998.

9. Ohno T. Fluorescence Inner-Filtering Correction for Determining the Humification Index of Dissolved Organic Matter. Environ Sci Technol. 2002;36: 742–746. doi:10.1021/es0155276

10. Miller MP, Simone BE, McKnight DM, Cory RM, Williams MW, Boyer EW. New light on a dark subject: comment. Aquatic Sciences. 2010;72: 269–275.

11. Lakowicz JR. Principles of fluorescence spectroscopy. Springer science & business media; 2013.

12. Hansen AM, Fleck J, Kraus TEC, Downing BD, von Dessonneck T, Bergamaschi B. Procedures for using the Horiba Scientific Aqualog® fluorometer to measure absorbance and fluorescence from dissolved organic matter. Reston, VA: U.S. Geological Survey; 2018. Report No.: 2018–1096. Available: https://doi.org/10.3133/ofr20181096

13. U.S. Geological Survey. National Water Information System data available on the World Wide Web (USGS Water Data for the Nation). In: USGS Surface-Water Annual Statistics for Wisconsin [Internet]. 2016. Available: https://waterdata.usgs.gov/nwis/uv/?referred_module=sw

14. U.S. Geological Survey. USGS Water-Quality Data for the Nation: Web Interface. 2016 [cited 11 Oct 2016]. Available: https://doi.org/10.5066/F7P55KJN
